# Contribution of cystatin C- and creatinine-based definitions of chronic kidney disease to cardiovascular risk assessment in 20 population-based and 3 disease cohorts: the BiomarCaRE project

**DOI:** 10.1186/s12916-020-01776-7

**Published:** 2020-11-09

**Authors:** Dietrich Rothenbacher, Martin Rehm, Licia Iacoviello, Simona Costanzo, Hugh Tunstall-Pedoe, Jill J. F. Belch, Stefan Söderberg, Johan Hultdin, Veikko Salomaa, Pekka Jousilahti, Allan Linneberg, Susana Sans, Teresa Padró, Barbara Thorand, Christa Meisinger, Frank Kee, Amy Jayne McKnight, Tarja Palosaari, Kari Kuulasmaa, Christoph Waldeyer, Tanja Zeller, Stefan Blankenberg, Wolfgang Koenig, on behalf of the BiomarCaRE consortium

**Affiliations:** 1 Institute of Epidemiology and Medical Biometry, Ulm University, Helmholtzstr. 22, 89081 Ulm, Germany; 2 Division of Clinical Epidemiology and Aging Research C070, German Cancer Research Center (DKFZ), Heidelberg, Germany; 3 Department of Epidemiology and Prevention, IRCCS Neuromed, Pozzilli, Italy; 4 Research Center in Epidemiology and Preventive Medicine (EPIMED), Department of Medicine and Surgery, University of Insubria, Varese, Italy; 5 Cardiovascular Epidemiology Unit, Institute of Cardiovascular Research, University of Dundee, Dundee, UK; 6 Vascular Medicine Unit, Institute of Cardiovascular Disease, University of Dundee, Dundee, UK; 7 Department of Public Health and Clinical Medicine, Umeå University, Umeå, Sweden; 8 Department of Medical Biosciences, Clinical Chemistry, Umeå University, Umeå, Sweden; 9 Finnish Institute for Health and Welfare, Helsinki, Finland; 10 Center for Clinical Research and Prevention, Bispebjerg and Frederiksberg Hospital, The Capital Region, Copenhagen, Denmark; 11 Catalan Department of Health, 08005 Barcelona, Spain; 12 Cardiovascular ICCC-Program, Research Institute Hospital de la Santa Creu i Sant Pau, IIB-Sant Pau, Barcelona, Spain; 13 Institute of Epidemiology, Helmholtz Zentrum München, German Research Center for Environmental Health, Neuherberg, Germany; 14 Independent Research Group Clinical Epidemiology, Helmholtz Zentrum München, German Research Center for Environmental Health, Neuherberg, Germany; 15 Ludwig-Maximilians-Universität München, Chair of Epidemiology at UNIKA-T Augsburg, Augsburg, Germany; 16 Queen’s University of Belfast, UK Clinical Research Collaboration Centre of Excellence for Public Health, Belfast, UK; 17 Centre for Public Health, School of Medicine, Dentistry and Biomedical Sciences, Queen’s University of Belfast, Belfast, UK; 18 Clinic for General and Interventional Cardiology, University Heart Center Hamburg, Hamburg, Germany; 19 German Center for Cardiovascular Research (DZHK e.V.), partner site Hamburg, Lübeck, Kiel, Hamburg, Germany; 20 Deutsches Herzzentrum München, Technische Universität München, Munich, Germany; 21 DZHK (German Centre for Cardiovascular Research), partner site Munich Heart Alliance, Munich, Germany

**Keywords:** Cohort study, Chronic kidney disease, Estimated glomerular filtration rate, Adverse outcome, Creatinine, Cystatin C

## Abstract

**Background:**

Chronic kidney disease has emerged as a strong cardiovascular risk factor, and in many current guidelines, it is already considered as a coronary heart disease (CHD) equivalent. Routinely, creatinine has been used as the main marker of renal function, but recently, cystatin C emerged as a more promising marker. The aim of this study was to assess the comparative cardiovascular and mortality risk of chronic kidney disease (CKD) using cystatin C-based and creatinine-based equations of the estimated glomerular filtration rate (eGFR) in participants of population-based and disease cohorts.

**Methods:**

The present study has been conducted within the BiomarCaRE project, with harmonized data from 20 population-based cohorts (*n* = 76,954) from 6 European countries and 3 cardiovascular disease (CVD) cohorts (*n* = 4982) from Germany. Cox proportional hazards models were used to assess hazard ratios (HRs) for the various CKD definitions with adverse outcomes and mortality after adjustment for the Systematic COronary Risk Evaluation (SCORE) variables and study center. Main outcome measures were cardiovascular diseases, cardiovascular death, and all-cause mortality.

**Results:**

The overall prevalence of CKD stage 3–5 by creatinine- and cystatin C-based eGFR, respectively, was 3.3% and 7.4% in the population-based cohorts and 13.9% and 14.4% in the disease cohorts. CKD was an important independent risk factor for subsequent CVD events and mortality. For example, in the population-based cohorts, the HR for CVD mortality was 1.72 (95% CI 1.53 to 1.92) with creatinine-based CKD and it was 2.14 (95% CI 1.90 to 2.40) based on cystatin-based CKD compared to participants without CKD. In general, the HRs were higher for cystatin C-based CKD compared to creatinine-based CKD, for all three outcomes and risk increased clearly below the conventional threshold for CKD, also in older adults. Net reclassification indices were larger for a cystatin-C based CKD definition. Differences in HRs (between the two CKD measures) in the disease cohorts were less pronounced than in the population-based cohorts.

**Conclusion:**

CKD is an important risk factor for subsequent CVD events and total mortality. However, point estimates of creatinine- and cystatin C-based CKD differed considerably between low- and high-risk populations. Especially in low-risk settings, the use of cystatin C-based CKD may result in more accurate risk estimates and have better prognostic value.

## Background

Chronic kidney disease (CKD) represents a global public health problem and affects a large proportion of the adult population worldwide [1, 2]. CKD has a complicated relationship with diabetes and hypertension and other associated diseases, and it is an independent risk factor for cardiovascular diseases (CVDs) as well as for all-cause mortality [1]. Outcomes of CKD include not only progression to end-stage renal disease (ESRD) but also complications such as hypertension, malnutrition, anemia, bone disease, and a decreased quality of life [3, 4].

Also, subclinical CKD has been associated with a large burden of disease and mortality [5]. This finding is clinically important because early detection and treatment of CKD can prevent or delay the progression of CKD and its adverse health outcomes [6]. Meanwhile, it has been demonstrated that the addition of a cystatin C-based equation improves overall risk classification for death, cardiovascular disease, and end-stage renal disease [7]. However, different equations, based on creatinine or cystatin C measurements, for estimating CKD seem to have different performance characteristics in high-risk and low-risk populations and subgroups such as older adults or patients with diabetes [8, 9]. An analysis of the clinical value in specific populations (e.g., for risk prediction) such as high-risk and low-risk CVD populations, older adults, or patients with diabetes would further help to assess the performance of the various estimated glomerular filtration rate (eGFR) estimation equations.

The aim of the study was to assess the prevalence of CKD using creatinine (Cr)- and cystatin C (cysC)-based eGFR equations and their comparative risks for cardiovascular and mortality in participants of cohorts of the MORGAM/BiomarCaRE consortium representing the general population and cohorts with manifest CVD. We also compared the strength of the associations and prognostic values between population-based general and disease cohorts and in specific subgroups (e.g., older adults, sex, participants with hypertension and diabetes).

## Methods

### Study populations and study design

The present study has been conducted within the MORGAM/BiomarCaRE projects, described in detail previously [10, 11], with harmonized data from 20 population-based cohorts from 7 European countries and 3 CVD cohorts from Germany. The harmonized data variables included baseline information on sex, age, smoking status, hypertension (defined as systolic blood pressure > 140 mmHg or anti-hypertensive medication), and diabetes (defined as self-report or antidiabetic medication). In addition, total cholesterol, C-reactive protein (CRP), N-terminal pro-B-type natriuretic peptide (Nt-proBNP), and troponin I were included, as well as study outcome information (details below). Details of the studies are included in Additional file 1, Table S1-S3, Box S1 [12–28] and also can be found elsewhere [10, 11, 29].

### Laboratory measurements

In the population-based cohorts of the MORGAM/BiomarCaRE study, creatinine was measured with the kinetic alkaline picrate Jaffe method with the isotope dilution mass spectrometry (IDMS) traceable (NIST SRM 967) Abbott Architect Assay CREATININE on the Architect c8000. Cystatin C was measured with the immunoassay cystatin C on an Abbott Diagnostics ARCHITECT. All analyses were done at the BiomarCaRE central laboratory at Mainz and after the move in the Medical University Center Hamburg-Eppendorf in Hamburg. The intra- and inter-assay coefficients of variation (CVs) were measured using samples of medium concentrations (creatinine: medium = 1.39–2.44 mg/dL, high = 2.25–7.3 mg/dL; cystatin C: high = 2.95–4.77 mg/L). The intra-assay CVs for creatinine ranged from 0.09 to 5.2% and for cystatin C from 0.78 to 4.0%, respectively. The inter-assay CV for creatinine ranged from 2.3 to 8.1% and for cystatin C from 1.8 to 12.5% for the measurements in the population-based cohorts. In the disease cohorts, creatinine was measured locally immediately by standardized routine methods in the respective laboratories of the participating centers.

In addition, total cholesterol was measured locally by routine methods and subject to a central quality control in the general population cohorts (details under https://www.thl.fi/publications/monica/tchol/tcholqa.htm). C-reactive protein was measured on an Abbott Architect c8000 system and the CRP Vario immunoassay (intra-assay CV 0.87–3.79%, inter-assay CV 2.57–4.71%, using samples of low concentration (2.8–4.2 mg/L)). N-terminal pro-B-type natriuretic peptide (NT-proBNP) levels were measured on an ELECSYS 2010 or a Cobas e411 using an electrochemiluminescence immunoassay (ECLIA, Roche Diagnostics) (intra-assay CV 1.48–7.04%, inter-assay CV 4.74–9.18% using samples of low concentration (115.6–166.4 pg/mL)).

### Assessment of chronic kidney disease

Kidney function was assessed by means of eGFR based on the latest equations from the Chronic Kidney Disease Epidemiology Collaboration (CKD-EPI), and included creatinine and cystatin C [7].

*eGFRcrea according to the Chronic Kidney Disease Epidemiology Collaboration (CKD-EPI*_*crea*_*) equation* [30]: eGFR = 141 × min (Cr/*k*, 1)^*a*^ × max (Cr/*k*, 1)^−1.209^ × (0.993)^age^ × (1.018 if female) × (1.159 if black) where *k* is 0.7 for females and 0.9 for males, *a* is − 0.329 for females and − 0.411 for males, min indicates the minimum of Cr/*k* or 1, and max indicates the maximum of Cr/*k* or 1.

*Cystatin C-based eGFR according to CKD-EPI collaboration* [31]: eGFR (CKD-EPI_cysC_) = 127.7 × (cysC)^−1.17^ × age^−0.13^ × (0.91 if female) × (1.06 if black).

CKD stage 3–5 was defined as eGFR of less than 60 mL/min/1.73 m^2^. In equations, Cr is given in mg/dL, cysC in mg/L, age in years, weight in kg, and eGFR in mL/min/1.73 m^2^. Only participants with measurements of creatinine and cystatin C were included in the final statistical analysis.

### Outcome definitions

The following outcomes were included in the analysis: (1) cardiovascular mortality (fatal myocardial infarction, fatal stroke, cardiac death, unclassified death) and (2) cardiovascular disease which was defined in the population-based studies as the first fatal or non-fatal coronary heart disease event or cerebral infarction. The coronary event included acute definite or possible myocardial infarction or coronary death, unstable angina pectoris, cardiac revascularization, or unclassifiable death. Definition of CVD disease outcomes was based on data harmonized in the MORGAM project. For population-based cohorts, participants with prevalent CVD at baseline were excluded from this outcome analysis (note that these participants are still included in the supplementary tables). For the disease cohorts, subsequent CVD was defined as CVD as the main cause of death and non-fatal stroke or myocardial infarction. (3) Total mortality as an endpoint was defined as death due to any cause during the follow-up time. More details of the event classification are provided elsewhere [11, 32] and in the MORGAM manual [29]. The follow-up started at the date of baseline examinations. Duration of follow-up in each cohort is described in Additional file 1, Table S1.

### Statistical analysis

The study populations were described with respect to baseline sociodemographic and medical characteristics. The prevalence of CKD was calculated and displayed across specific age categories. Cox proportional hazards models were used to assess hazard ratios (HRs) for the various CKD definitions with adverse cardiovascular outcomes and mortality after adjustment for the SCORE variables (age, sex, smoking status, systolic blood pressure, total cholesterol) [33, 34] and also study cohort. The proportional hazards assumption was checked graphically and based on the multivariable model. Besides overall results, analyses were also done according to age (< 65, ≥ 65 years), sex, history of hypertension, and diabetes at baseline. In addition, the area under the curve (AUC) with 95% CI and the net reclassification improvement (NRI) [35] for events and non-events by adding creatinine-based and cystatin C-based CKD to the ESC score variables’ adjusted model were calculated according to the risk strata of < 1%, 1 to < 5%, 5 to < 10, and ≥ 10% of estimated 10-year risk for the various events. Finally, we used a restricted cubic spline regression with 3 degrees of freedom for multivariate analysis within the context of the Cox proportional hazards models. SAS version 9.4 (SAS Institute Inc., Cary, NC) and R version 3.5.1 (R Foundation for Statistical Computing) were used for all analyses. We used our own routines in SAS and R as well as the R package “rms” for spline regression modeling. We ran most of the analytical steps in both programs, underpinned by the 4-eye principle, to assure the quality of the results.

## Results

A total of 20 population-based cohorts with 75,367 participants (median age 50 years, 50.9% men, 4.4% diabetes) and with an average follow-up between 2.82 and 23.47 years were included in the study. In addition, three disease cohorts with 4982 patients with manifest CVD (median age 63 years, 75.6% men, 18.7% diabetic) and an average follow-up time between 0.47 and 9.37 years, respectively, were available within the MORGAM/BiomarCaRE consortium for this analysis (for details, see Table 1). Further details of the included cohorts, their main baseline characteristics, and details of renal function are displayed in Additional file 1, Tables S1-S3. In the population-based cohorts, the incidence of cardiovascular disease (non-fatal and fatal CVD events combined), cardiovascular mortality, and total mortality, respectively, was 8.2, 4.2, and 10.9 per 1000 person years, whereas it was 21.2, 6.9, and 17.9 in the diseased cohorts, respectively (Table 1).

**Table 1:** Baseline characteristics of the study populations

	Population-based cohorts	Disease cohorts
Number of cohorts, *n*	20	3
Number of subjects, *n*	75,367	4982
Men, *n* (%)	38,350 (50.9%)	3766 (75.6%)
Age at baseline, years		
Median (Q1, Q3)	50.0 (41.0, 59.0)	63.0 (54.0, 69.0)
Proportion ≥ 65 years	13.1%	42.6%
Daily smokers, *n* (%)	24,077 (31.9%)	892 (17.9%)
Diabetes, *n* (%)	3286 (4.4%)	930 (18.7%)
Hypertension, *n* (%)	30,811 (40.9%)	3514 (70.5%)
Body mass index (kg/m^2^), mean (SD)	26.9 (4.6)	27.6 (4.1)
Total cholesterol (mmol/L)*	5.7 (5.0, 6.5)	4.9 (4.1, 5.7)
CRP (mg/L)*	1.3 (0.6, 2.9)	1.5 (0.4, 5.1)
Nt-proBNP (pg/mL)*	45.9 (24.2, 86.3)	308.0 (116.0, 803.0)
Troponin I (ng/L)*	2.3 (1.4, 3.7)	10.4 (4.6, 29.7)
eGFR (mL/min/1.73m^2^)*		
CKD-EPI_crea_	97.6 (85.9, 107.6)	82.7 (68.7, 94.6)
CKD-EPI_cysC_	92.5 (76.4, 111.5)	91.1 (69.1, 116.2)
CKD stage 3+, *n* (%)		
CKD-EPI_crea_	2450 (3.3%)	691 (13.9%)
CKD-EPI_cysC_	5562 (7.4%)	719 (14.4%)
Endpoints (*n*, incidence rate per 1000 person years (95% CI))		
Cardiovascular disease	6850, 8.2 (95% CI 8.0–8.4)	371, 21.2 (95% CI 19.2–23.5)
Cardiovascular mortality	3796, 4.2 (95% CI 4.1–4.3)	132, 6.9 (95% CI 5.8–8.2)
Total mortality	9840, 10.9 (95% CI 10.6–11.1)	343, 17.9 (95% CI 16.1–19.9)

*Median (interquartile range, Q1, Q3)

The distribution of the eGFR as calculated with measurements of creatinine and cystatin C is shown in Additional file 1, Fig. S1 for the population-based cohorts in total (panel A) and also stratified according to age (Fig. S1 panels B and C) and in Additional file 1, Fig. S2 for the disease cohorts, also in total and stratified according to age. Notably, especially in the population-based cohorts, the distribution in the older population (Fig. S1 panel C) is quite different between the two eGFR formulas.

**Fig. 1:**
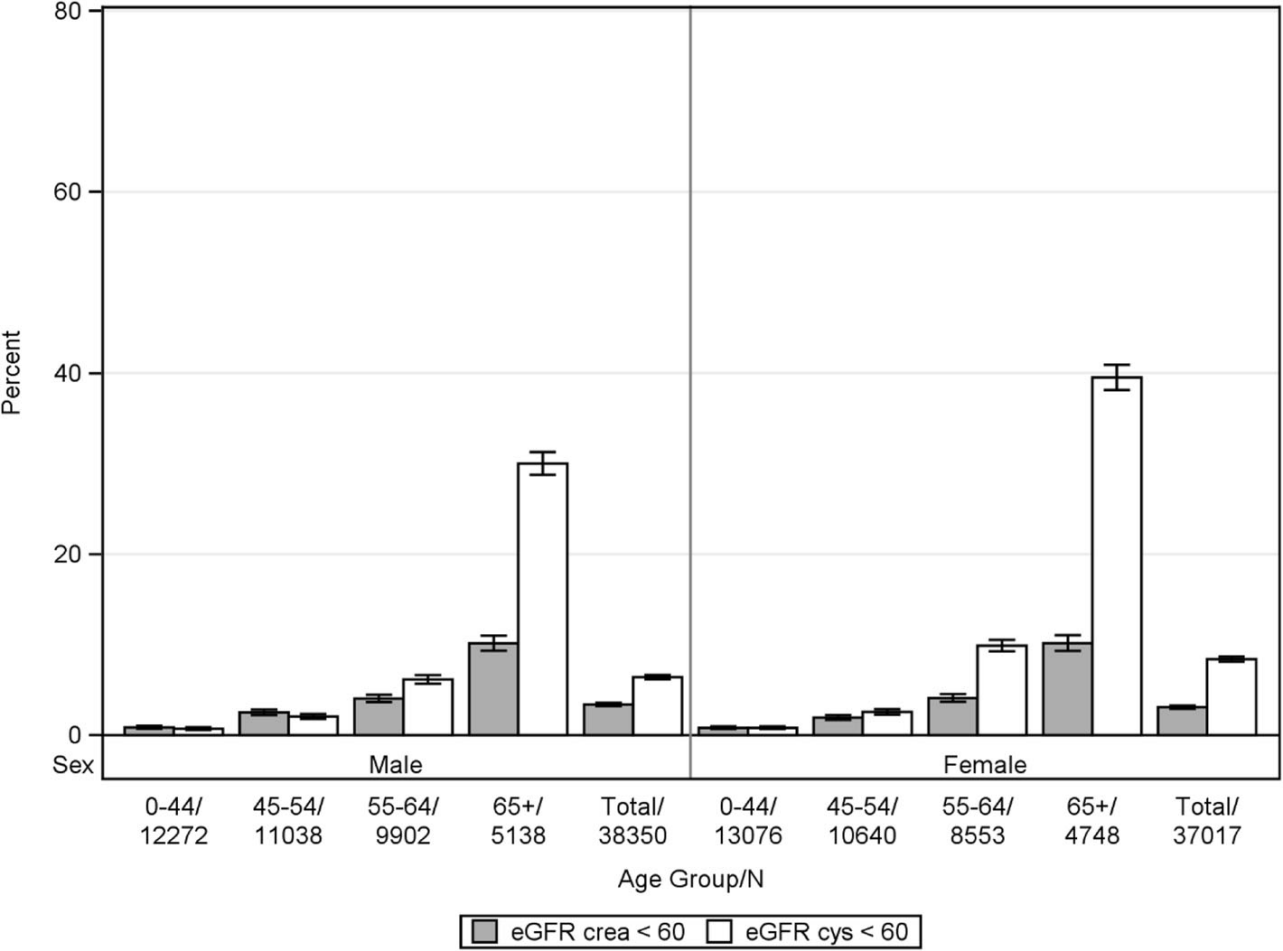
Prevalence of CKD based on different eGFR estimating equations in the population-based cohorts (histograms represent prevalence in % and bars 95% CIs)

**Fig. 2:**
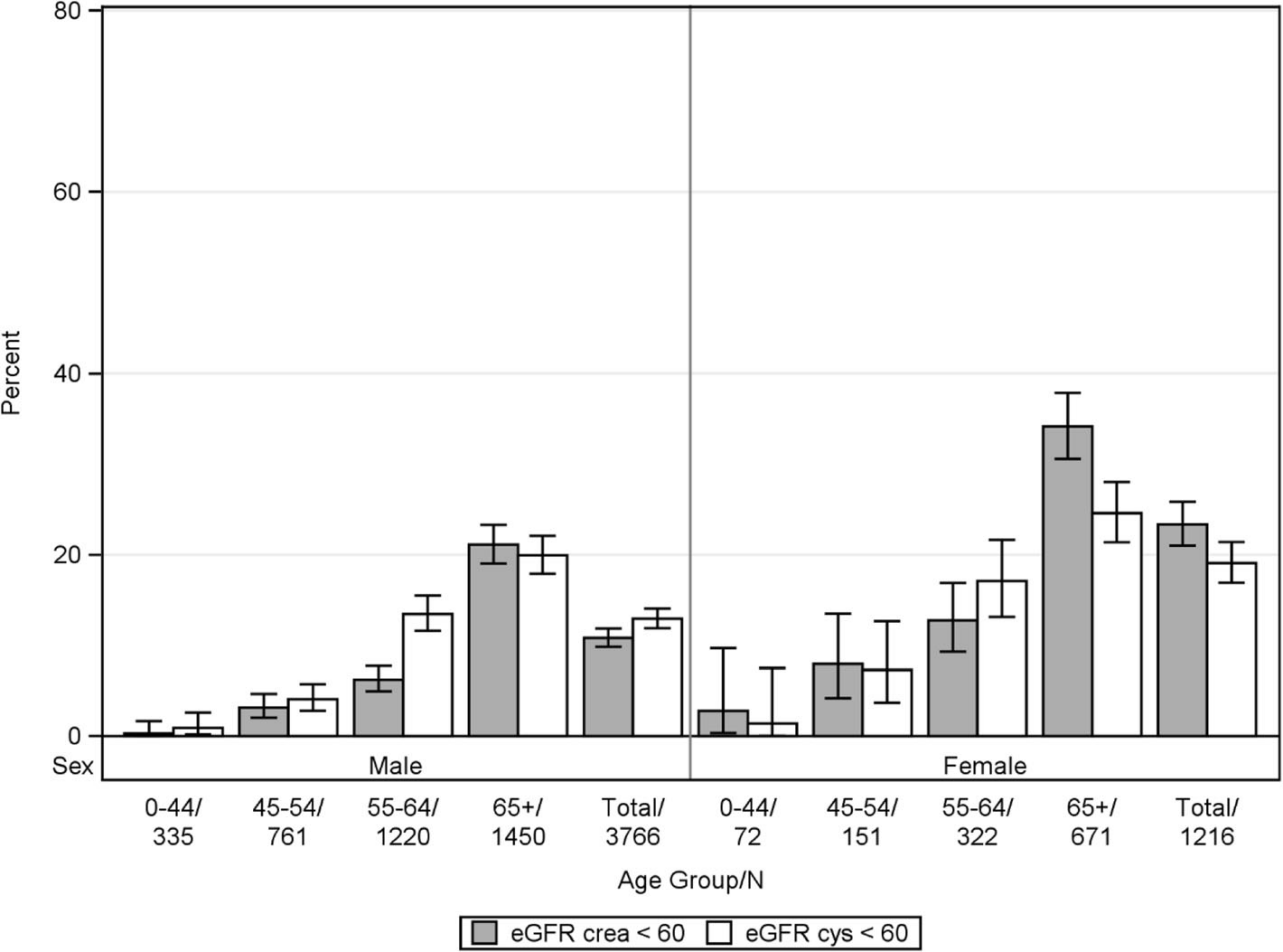
Prevalence of CKD based on different eGFR estimating equations in the disease cohorts (histograms represent prevalence in % and bars 95% CIs)

The overall prevalence of CKD stage 3–5 by CKD-EPI_crea_ and CKD-EPI_cys_ eGFR, respectively, was 3.3% and 7.4% in the population-based cohorts and 13.9% and 14.4% in the disease cohorts. In males and females, the prevalence of CKD showed a steep increase with age. In the population-based cohorts, prevalence was higher based on CKD-EPI_cysC_ (Fig. 1), whereas patterns were different based on CKD-EPI in the disease cohorts (Fig. 2).

**Fig. 3:**
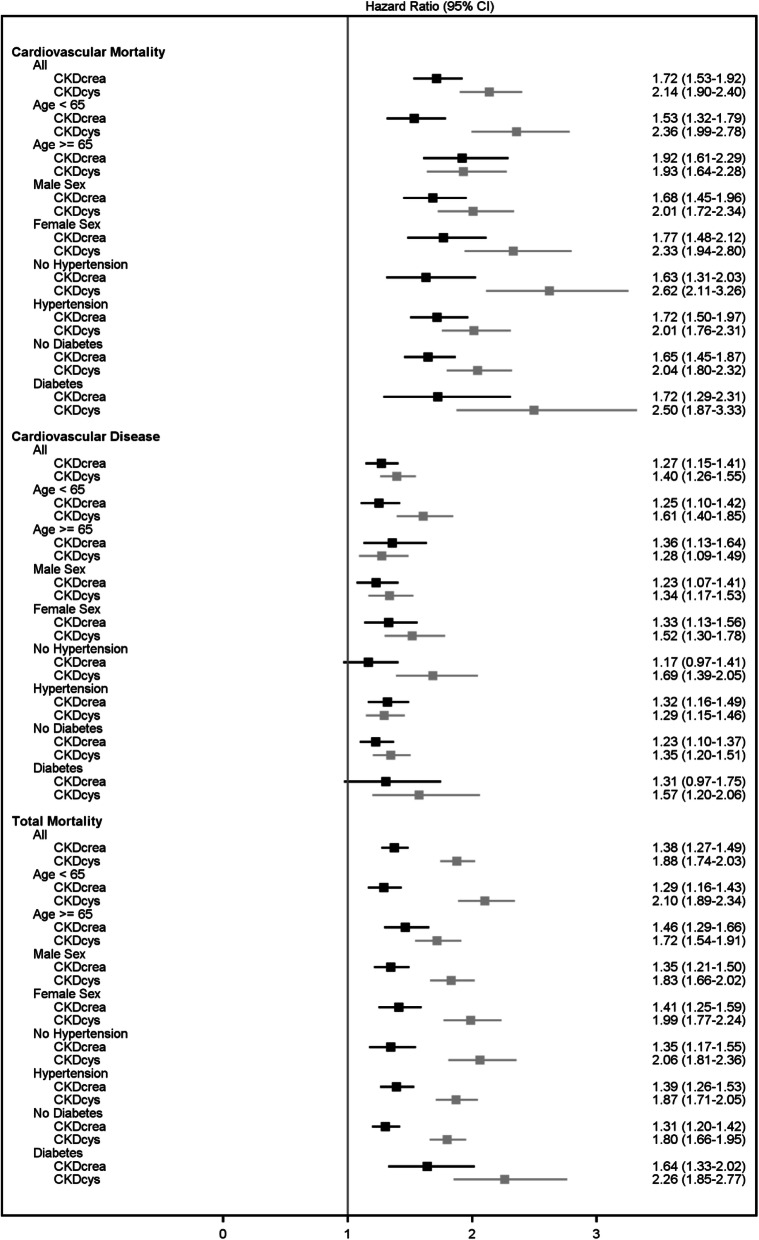
Association of CKD with various endpoints in population-based cohorts (squares represent HR and 95% CIs)

Figure 3 shows the association (HR and 95% CI) of both CKD-EPI equations with CVD mortality, fatal and non-fatal CVD events, and total mortality after adjustment for the ESC score variables for the population-based cohorts in total, and stratified according to age (cut point 65 years), sex, and hypertension and diabetes. The area under the curve (AUC) and net reclassification index (NRI) for events and non-events are also provided (details in Additional file 1, Tables S4 for population-based cohorts). For example, the HR for CVD mortality was 1.72 (95% CI 1.53 to 1.92) with CKD-EPI_crea_ and it was 2.14 (95% CI 1.90 to 2.40) based on CKD-EPI_cysC_ after adjustment for covariates. In general, the HRs were higher for cystatin C-based eGFR compared to creatinine-based eGFR for all three outcomes, especially evident for total mortality. Mainly, the NRI_ne_ was always larger for CKD-EPI_cysC_ for all three outcomes. It was notably high for the age strata ≥ 65 years for cardiovascular mortality and total mortality (4.2% and 6.5%, respectively) and highest for the stratum diabetes for total mortality (NRI_ne_ 9.4%).

**Fig. 4:**
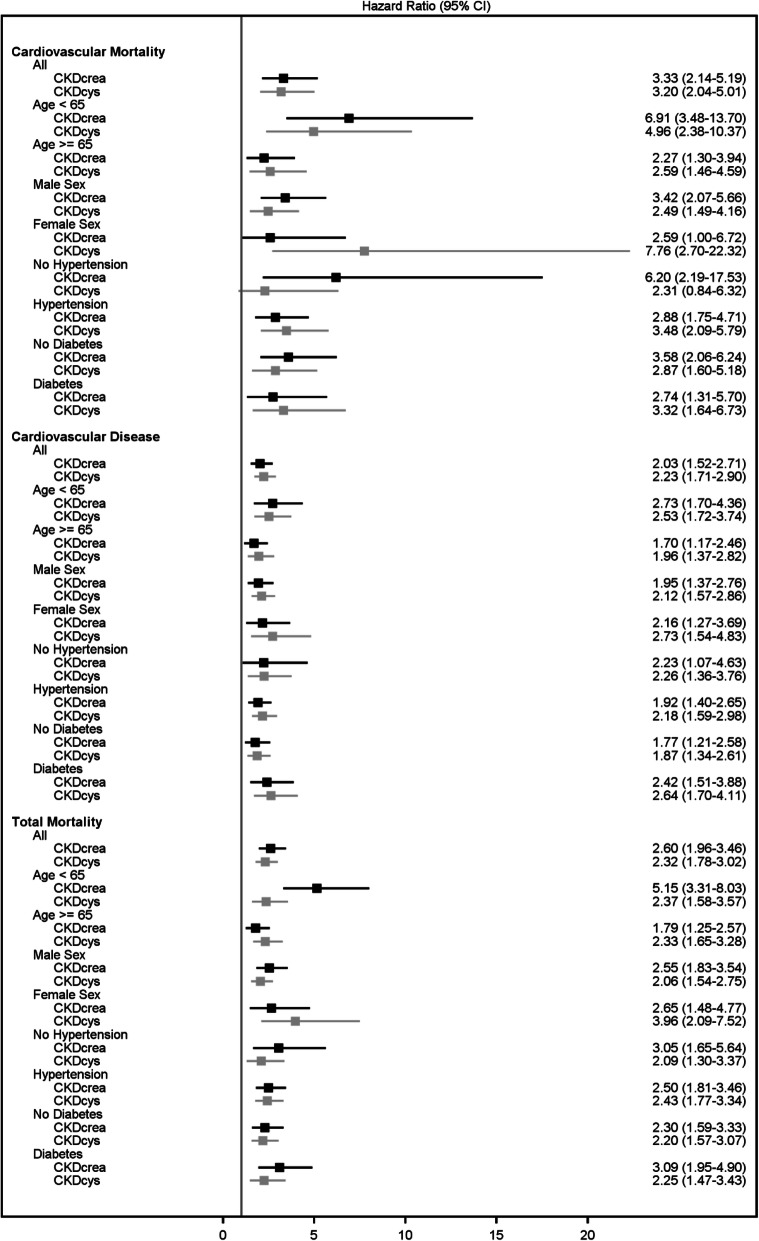
Association of CKD with various endpoints in disease cohorts (squares represent HR and 95% CIs)

Figure 4 shows the association of CKD with the various outcomes in the diseased cohorts (details in Additional file 1, Table S5 for disease cohorts). For example, the HR for CVD mortality was 3.33 (95% CI 2.14 to 5.19) with CKD-EPI_crea_ and 3.20 (95% CI 2.04 to 5.01) based on CKD-EPI_cysC_ after adjustment for covariates. Especially for mortality, HRs were more often higher for CKD-EPI_crea_. For both CKD variables in disease cohorts, NRI_ne_ was highest in the strata female sex, but the patterns were less clear than those in the population-based cohorts.

Details of reclassification in both types of cohorts are shown in Additional file 1, Table S6. In the population-based cohorts, a large proportion (47.4%) of the CKD-crea-defined cases were re-graded by the CKD-cysC-defined CKD to no CKD. The pattern, in general, was similar to the diseased cohorts, although the much lower numbers of the disease cohorts have to be considered.

**Fig. 5:**
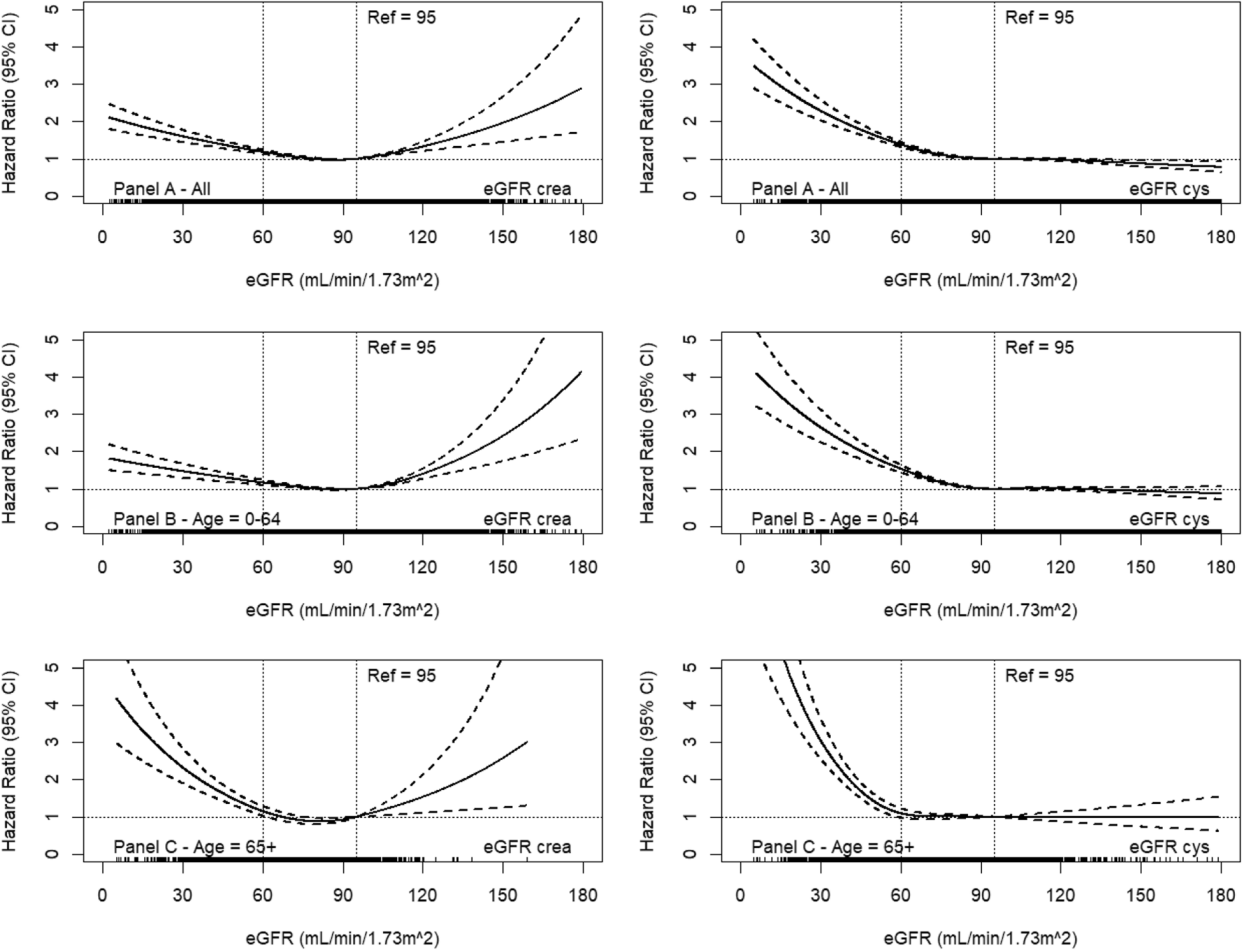
Spline regression representing creatinine (left side)- and cystatin C (right side)-based eGFR associated with hazard ratio (HR) for mortality after adjustment for SCORE variables and study cohort for all and after stratification for age in the population-based cohorts (for details, see the “Methods” section)

Figure 5 shows adjusted HR for creatinine-based eGFR (left side) and for cystatin C-based eGFR (right side) for total mortality in the population-based cohorts overall (panel A) and after stratification for age (panel B age up to 64 years, panel C age 65+ years). Although the increase of the HR with decreasing eGFR is steeper with the cystatin C-based eGFR, this difference is mainly driven by the trend in the population up to 65 years. Furthermore, a clear increase of the HRs is evident below 60 mL/min/1.73m^2^, even in the age strata 65+ years, pointing to the validity of the current threshold for CKD stage 3+. Additional file 1, Fig. S7 shows the results for mortality in the disease cohorts showing similar patterns for creatinine- and cystatin C-based eGFR. Additional file 1, Fig. S8 shows the AUC for cystatin C- and creatinine-based eGFR with consistently higher AUC values for the cystatin C-based eGFR definition in both cohorts in all strata.

## Discussion

This large cohort study conducted within the framework of the MORGAM/BiomarCaRE consortium clearly demonstrated that CKD is an important risk factor for subsequent CVD events and total mortality, both in low- as well as high-risk populations. However, especially in the (low-risk) population-based cohorts, it was evident that analysis of the creatinine- and cystatin C-based eGFRs and their consequences differed considerably. Cystatin C-defined CKD showed a higher prevalence especially in older adults, and cystatin C-based eGFR was associated with higher CVD risk estimates. It seemed also to be associated with better risk classification, especially evident for mortality in older adults. Notably, this difference was not obvious in the relatively older, high-risk disease cohorts. Furthermore, we found evidence that the conventional threshold of CKD (60 mL/min./1.73m^2^) seems valid also for participants aged 65 years and older, although the increase in the estimated risk was less steep compared to the younger participants. Notably, higher eGFR-creatinine (≥ 90 mL/min/1.73 m2) was associated with higher hazard ratio of adverse events, while higher eGFR-cysC was not. Compared to a creatinine-based definition of CKD, the cystatin C-based definition of CKD seemed to have a higher specificity, a measure especially important in populations with low prevalence of CKD and therefore resulted in many fewer false positives.

### Prevalence of CKD and differences of eGFRs

Based on a recent review, the prevalence of CKD stage 3–5 varied considerably in studies from 19 general populations from 13 European countries and was between 1.0 and 5.9% in the adult population. Many factors including measurement issues certainly contribute to this variation besides differences in comorbidity [36]. These numbers are in line with our estimates in the population-based cohorts. Estimates of cystatin C CKD were in general slightly higher, but striking differences occurred especially in the older adults. In the population-based cohorts, cystatin C-based CKD prevalence was much higher in older adults, whereas in the diseased cohorts CKD-EPI_crea_ resulted in a higher CKD prevalence in participants aged 65 years or older, especially in females.

Cystatin C is more sensitive than creatinine especially in the detection of early kidney dysfunction among various patient groups such as diabetes, in sarcopenia, and also in the older adults. Potential confounders for creatinine-based eGFR are muscle mass, dehydration, dietary factors, and other comorbid diseases. As suggested in other studies that have included older adults, the creatinine-based definition of CKD in high-risk groups may result in a higher prevalence estimate because of the association of creatinine levels with many comorbid conditions such as muscle mass, frailty, or diabetes [37, 38]. Cystatin C-based eGFR is less influenced by age or ethnicity, but other factors such as obesity, inflammation, and smoking as well as intake of glucocorticoids may affect serum values.

### Differences in risk estimation for various outcomes

Our results show that in middle-aged low-risk populations, cystatin C-based CKD classification had a stronger association with risk of CVD morbidity and especially mortality, as seen in other studies [7, 39, 40]. CKD should be considered as an equally relevant risk factor for mortality and end-stage renal disease for patients with, as well as those without, hypertension. Notably, associations were even stronger in patients without hypertension [41]. For patients with diabetes, the relative risks were much the same as for non-diabetic persons, pointing to the importance of CKD for adverse outcomes [34]. As suggested in other studies, in patients with diabetes, the use of a cystatin C-based CKD definition offered better clinical utility for risk prediction than creatinine-based equations [8]. In addition, a systematic review including 23 studies came to the conclusion that cystatin C-based eGFR represents measured GFR well in patients with diabetes [9]. The observation in our spline plots that higher creatinine-based eGFR again showed an adverse association with the outcomes has been shown previously [7, 42]. It probably reflects the associations with comorbid conditions such as lower muscle mass, frailty, or diabetes and implies that creatinine-based eGFR, especially in elderly, has severe limitations, particularly when it exceeds 100 mL/min/1.73m^2^ [43]. Thus, it should be investigated further as it was also very obvious in subjects aged 64 or younger in our analysis.

Furthermore, especially in younger and low-risk populations, the cystatin C-based eGFR may deliver more valid results [7] compared to creatinine-based eGFR, an observation in line with our data. In high-risk populations or older adults, this may be different [44]. However, evidence from studies including older adults is so far inconsistent. A study including 1639 British men aged 71 to 92 years [45], a study including 1741 participants with CKD and a mean age of 73 years [44], and a study conducted in older adult women (mean age 75.2 years) [46] showed no benefit from using cystatin C-based eGFR equations when compared to creatinine-based ones. However, the large study of Shlipak and colleagues, including 11 cohorts of the general population (with a mean age of 60 years) and 5 cohorts with CKD (mean age 55 years), found that cystatin C-based eGFR could better categorize risk than creatinine-based eGFR, especially around the threshold of 60 mL/min/1.73m^2^ [7], in line with recommendations of KDIGO [5]. However, the younger age in that study has to be considered.

### Implications

All methods to estimate GFR are associated with systematic and random error; however, on a positive note, they have less time-to-time variability than measured GFR [47]. Furthermore, it is important to consider the underlying risk profile of the population on the impact of the relative risk estimates. Especially in low-risk settings, cystatin C-based eGFR seems more accurate and delivers fewer false positives. The lower risk estimates associated with CKD in the diseased cohorts probably reflect the higher prevalence of comorbid conditions and the higher baseline risk within this population. As suggested by some authors, both CKD-EPI equations may have similar accuracy but show bias in opposite directions and the combination of both may deliver the best results in older adults [48, 49]. However, currently, measurement of cystatin C is more expensive than creatinine. Therefore, further research should focus on the cost-effectiveness of cystatin C-based measurements of CKD, especially in low-risk settings and older populations with the aim of finding ways to reduce the costs of measurement within routine medical care.

### Strength and limitations

We included a large number of general population-based studies which had been assembled within the MORGAM/BiomarCaRE consortium, used harmonized data, and could rely on centralized measurements for creatinine and cystatin C. However, unfortunately, cystatin C measurements had not been standardized to the global WHO reference material, which however should not affect internal validity of results, but external comparability. We also had cohorts of patients with prevalent CVD available, although the numbers of included participants were much lower compared to the population-based studies and they only came from Germany. Unfortunately, the laboratory measures of the disease cohorts had been measured at each study center. Furthermore, we relied on one single measurement to define CKD, and therefore, some of the participants would not have been classified as having CKD if measured twice and if a time period of 6 months had been applied to define the chronicity of CKD. Furthermore, we could not include measurements of protein or albumin in urine as they were not available in a standardized manner in all included studies. Proteinuria or albuminuria is indeed an import prognostic factor and used as an indicator for renal damage especially in CKD stages 1 and 2 and explains much of the risk increase within these stages. However, we dichotomized CKD and summarized all stages 3 and above versus the rest, according to the clinically used threshold.

## Conclusions

CKD is an important risk factor for subsequent CVD events and total mortality. However, point estimates of creatinine-based definition and cystatin C-based CKD differed considerably between low- and high-risk populations. Especially in low-risk settings, the use of cystatin C-based eGFR may result in more accurate risk estimates and have better prognostic value compared to creatinine-based CKD definition. Therefore, the clinical utility of both equations in different risk populations and risk groups has to be considered and should be evaluated further.

## Supplementary information

**Additional file 1: **The Additional file 1 contains additional descriptive and analysis results and information about the included studies. **Table S1.** List of included population-based and disease-cohorts. **Table S2.** Main baseline characteristics of population-based and disease-cohorts included. **Table S3.** Renal function at baseline of population-based and disease-cohorts included. **Figure S1.** Distribution of the estimated glomerular filtration rate (eGFR) based on creatinine and cystatin C in the population based cohorts. **Figure S2.** Distribution of the estimated glomerular filtration rate (eGFR) based on creatinine and cystatin C in the disease cohorts. **Table S4.** Association of CKD with various endpoints (HR and 95% CI) including area-under the curve (AUC with 95% CI) and net-reclassification index (NRI with 95% CI) in population-based cohorts. **Table S5.** Association of CKD with various endpoints (HR and 95% CI) including area-under the curve (AUC with 95% CI) and net-reclassification index (NRI with 95% CI) in disease cohorts. **Table S6.** Agreement of reclassification by the CKD-EPIcysC based definition compared to CKD-EPIcrea based definition in the population-based cohorts (Panel A) and in the disease cohorts (Panel B). **Figure S7.** Spline regression representing creatinine (left side) and cystatin C (right side) based eGFR associated with hazard ratio’s (HR) for mortality after adjustment for SCORE-variables and study center for all and after stratification for age in the disease cohorts. **Box S1.** Further Description of Study Cohorts.

## Data Availability

Due to ethical restrictions, the data cannot be made publicly available but may be available upon request. The request should be directed to Prof. Rothenbacher (dietrich.rothenbacher@uni-ulm.de).

## Abbreviations

AUC: Area under the ROC curve
BMI: Body mass index
CHD: Coronary heart disease
CI: Confidence interval
Cr: Creatinine
cysC: Cystatin C
CKD: Chronic kidney disease
CKD-EPI: Chronic Kidney Disease Epidemiology Collaboration
CV: Coefficient of variation
CVD: Cardiovascular disease
eGFR: Estimated glomerular filtration rate
ESRD: End-stage renal disease
hs-cTnT: High-sensitivity cardiac troponin T
hs-cTnI: High-sensitivity cardiac troponin I
hs-CRP: High-sensitivity C-reactive protein
HR: Hazard ratio
NT-proBNP: N-terminal pro-B-type natriuretic peptide
NRI: Net reclassification improvement
ROC: Receiver-operating characteristic
