# Supplementary material for: Contribution of cystatin C- and creatinine-based definitions of chronic kidney disease to cardiovascular risk assessment in 20 population-based and 3 disease cohorts: the BiomarCaRE project
Source: BMC Med. 2020 Nov 9;18:300. doi: 10.1186/s12916-020-01776-7 (PMC7650190; doi:10.1186/s12916-020-01776-7)
Supplement: Supplementary file 1 — Additional file 1: The Additional file 1 contains additional descriptive and analysis results and information about the included studies. Table S1. List of included population-based and disease-cohorts. Table S2. Main baseline characteristics of population-based and disease-cohorts included. Table S3. Renal function at baseline of population-based and disease-cohorts included. Figure S1. Distribution of the estimated glomerular filtration rate (eGFR) based on creatinine and cystatin C in the population based cohorts. Figure S2. Distribution of the estimated glomerular filtration rate (eGFR) based on creatinine and cystatin C in the disease cohorts. Table S4. Association of CKD with various endpoints (HR and 95% CI) including area-under the curve (AUC with 95% CI) and net-reclassification index (NRI with 95% CI) in population-based cohorts. Table S5. Association of CKD with various endpoints (HR and 95% CI) including area-under the curve (AUC with 95% CI) and net-reclassification index (NRI with 95% CI) in disease cohorts. Table S6. Agreement of reclassification by the CKD-EPIcysC based definition compared to CKD-EPIcrea based definition in the population-based cohorts (Panel A) and in the disease cohorts (Panel B). Figure S7. Spline regression representing creatinine (left side) and cystatin C (right side) based eGFR associated with hazard ratio’s (HR) for mortality after adjustment for SCORE-variables and study center for all and after stratification for age in the disease cohorts. Box S1. Further Description of Study Cohorts. [file 12916_2020_1776_MOESM1_ESM.docx]

**Table S1: List of included population-based and disease-cohorts**

| Cohort | Country | N | Years of baseline assessment | Mean follow-up time (yrs) | Creatinine measurements,  n (%) | Cystatin C measurements,  n (%) |
| --- | --- | --- | --- | --- | --- | --- |
| **Population-based cohorts** n=20 |  |  |  |  |  |  |
| MONICA/KORA S3 | Germany | 4692 | 1994-1995 | 13.11 | 4666 (99.4%) | 4665 (99.4%) |
| MONICA/KORA S4 | Germany | 4221 | 1999-2001 | 8.63 | 4221 (100.0%) | 3558 (84.3%) |
| Catalonia 86 | Spain | 2571 | 1986-1988 | 10.20 | 2393 (93.1%) | 2408 (93.7%) |
| Catalonia 90 | Spain | 2934 | 1990-1992 | 7.51 | 2731 (93.1%) | 2734 (93.2%) |
| FINRISK 97 | Finland | 5069 | 1997 | 13.11 | 4620 (91.1%) | 4615 (91.0%) |
| DAN-MONICA 1 | Denmark | 4052 | 1982-1984 | 23.01 | 3708 (91.5%) | 3714 (91.7%) |
| DAN-MONICA 2 | Denmark | 1504 | 1987-1988 | 21.79 | 1494 (99.3%) | 1494 (99.3%) |
| DAN-MONICA 3 | Denmark | 2026 | 1991-1992 | 17.00 | 2005 (99.0%) | 2005 (99.0%) |
| Moli-sani | Italy | 24325 | 2005-2010 | 4.26 | 23647 (97.2%) | 23666 (97.3%) |
| N-Sweden 86 | Sweden | 1625 | 1986 | 23.47 | 1601 (98.5%) | 1601 (98.5%) |
| N-Sweden 90 | Sweden | 1576 | 1990 | 20.57 | 1421 (90.2%) | 1421 (90.2%) |
| N-Sweden 94 | Sweden | 1893 | 1994 | 16.45 | 1861 (98.3%) | 1854 (97.9%) |
| N-Sweden 99 | Sweden | 1789 | 1999 | 12.20 | 1726 (96.5%) | 1725 (96.4%) |
| N-Sweden 4 | Sweden | 1863 | 2004 | 7.70 | 1810 (97.2%) | 1810 (97.2%) |
| N-Sweden 9 | Sweden | 1704 | 2009 | 2.82 | 1627 (95.5%) | 1627 (95.5%) |
| PRIME Belfast | UK | 2745 | 1991-1994 | 15.85 | 1856 (67.6%) | 1865 (67.9%) |
| SHHEC 1 | UK | 11573 | 1984-1987 | 21.13 | 10776 (93.1%) | 10744 (92.8%) |
| SHHEC 21 | UK | 1016 | 1989 | 18.54 | 680 (66.9%) | 680 (66.9%) |
| SHHEC 2 | UK | 1754 | 1992 | 15.06 | 1651 (94.1%) | 1651 (94.1%) |
| SHHEC 3 | UK | 1656 | 1995 | 13.61 | 1640 (99.0%) | 1640 (99.0%) |
| Total |  | 80588 |  | 12.10 | 76134 (94.5%) | 75477 (93.7%) |
| **Disease cohorts** n=3 |  |  |  |  |  |  |
| AtheroGene | Germany | 3476 | 1996-2004 | 4.49 | 3450 (99.3%) | 2162 (62.2%) |
| KAROLA | Germany | 1204 | 1999-2000 | 9.37 | 1152 (95.7%) | 1162 (96.5%) |
| StenoCardia | Germany | 1818 | 2007-2008 | 0.47 | 1809 (99.5%) | 1724 (94.8%) |
| Total |  | 6498 |  | 4.29 | 6411 (98.7%) | 5048 (77.7%) |

SHHEC=Scottish Heart Health Extended Cohort

**Table S2: Main baseline characteristics of population-based and disease-cohorts included**

| Cohort | Country | N | Men (%) | Age, yr (Mean, SD) | Age 65+, yr (%) | Daily smoking (%) | Diabetes (%) | Hypertension (%) |
| --- | --- | --- | --- | --- | --- | --- | --- | --- |
| **Population-based cohorts** n=20 |  |  |  |  |  |  |  |  |
| MONICA/KORA S3 | Germany | 4640 | 2336 (50.3%) | 49.9 (14.1) | 900 (19.4%) | 1316 (28.4%) | 237 (5.1%) | 1971 (42.5%) |
| MONICA/KORA S4 | Germany | 3558 | 1704 (47.9%) | 47.8 (13.8) | 548 (15.4%) | 946 (26.6%) | 163 (4.6%) | 1221 (34.3%) |
| Catalonia 86 | Spain | 2392 | 1166 (48.7%) | 45.7 (11.5) | 36 (1.5%) | 865 (36.2%) | 94 (3.9%) | 467 (19.5%) |
| Catalonia 90 | Spain | 2731 | 1626 (59.5%) | 44.2 (11.7) | 18 (0.7%) | 1173 (43.0%) | 114 (4.2%) | 414 (15.2%) |
| FINRISK 97 | Finland | 4591 | 2304 (50.2%) | 47.6 (13.2) | 523 (11.4%) | 1131 (24.6%) | 297 (6.5%) | 2148 (46.8%) |
| DAN-MONICA 1 | Denmark | 3708 | 1895 (51.1%) | 44.8 (11.0) | 0 | 2098 (56.6%) | 82 (2.2%) | 765 (20.6%) |
| DAN-MONICA 2 | Denmark | 1494 | 742 (49.7%) | 45.1 (11.1) | 0 | 720 (48.2%) | 18 (1.2%) | 255 (17.1%) |
| DAN-MONICA 3 | Denmark | 2005 | 1000 (49.9%) | 49.5 (14.2) | 397 (19.8%) | 965 (48.1%) | 67 (3.3%) | 571 (28.5%) |
| Moli-sani | Italy | 23646 | 11382 (48.1%) | 55.3 (11.9) | 5647 (23.9%) | 5313 (22.5%) | 1527 (6.5%) | 13266 (56.1%) |
| N-Sweden 86 | Sweden | 1601 | 814 (50.8%) | 45.2 (11.2) | 7 (0.4%) | 519 (32.4%) | 52 (3.2%) | 497 (31.0%) |
| N-Sweden 90 | Sweden | 1420 | 688 (48.5%) | 44.3 (11.3) | 0 | 442 (31.1%) | 32 (2.3%) | 409 (28.8%) |
| N-Sweden 94 | Sweden | 1853 | 911 (49.2%) | 49.1 (14.1) | 327 (17.6%) | 518 (28.0%) | 62 (3.3%) | 679 (36.6%) |
| N-Sweden 99 | Sweden | 1725 | 848 (49.2%) | 50.0 (14.0) | 339 (19.7%) | 356 (20.6%) | 68 (3.9%) | 700 (40.6%) |
| N-Sweden 4 | Sweden | 1810 | 883 (48.8%) | 50.2 (14.2) | 379 (20.9%) | 343 (19.0%) | 91 (5.0%) | 645 (35.6%) |
| N-Sweden 9 | Sweden | 1627 | 813 (50.0%) | 50.0 (14.1) | 304 (18.7%) | 267 (16.4%) | 78 (4.8%) | 597 (36.7%) |
| PRIME Belfast | UK | 1856 | 1856 (100.0%) | 54.2 (2.9) | 0 | 520 (28.0%) | 49 (2.6%) | 731 (39.4%) |
| SHHEC 1 | UK | 10739 | 5486 (51.1%) | 49.0 (7.5) | 21 (0.2%) | 4790 (44.6%) | 161 (1.5%) | 4064 (37.8%) |
| SHHEC 21 | UK | 680 | 313 (46.0%) | 46.5 (11.4) | 8 (1.2%) | 328 (48.2%) | 16 (2.4%) | 223 (32.8%) |
| SHHEC 2 | UK | 1651 | 792 (48.0%) | 51.6 (14.1) | 412 (25.0%) | 753 (45.6%) | 38 (2.3%) | 664 (40.2%) |
| SHHEC 3 | UK | 1640 | 791 (48.2%) | 45.3 (11.6) | 20 (1.2%) | 714 (43.5%) | 40 (2.4%) | 524 (32.0%) |
| Total |  | 75367 | 38350 (50.9%) | 50.3 (12.4) | 9886 (13.1%) | 24077 (31.9%) | 3286 (4.4%) | 30811 (40.9%) |
| **Disease cohorts** n=3 |  |  |  |  |  |  |  |  |
| AtheroGene | Germany | 2137 | 1667 (78.0%) | 61.7 (10.1) | 954 (44.6%) | 420 (19.7%) | 469 (21.9%) | 1637 (76.6%) |
| KAROLA | Germany | 1129 | 952 (84.3%) | 59.3 (8.1) | 356 (31.5%) | 59 (5.2%) | 200 (17.7%) | 616 (54.6%) |
| StenoCardia | Germany | 1716 | 1147 (66.8%) | 61.4 (13.5) | 811 (47.3%) | 413 (24.1%) | 261 (15.2%) | 1261 (73.5%) |
| Total |  | 4982 | 3766 (75.6%) | 61.0 (11.0) | 2121 (42.6%) | 892 (17.9%) | 930 (18.7%) | 3514 (70.5%) |

**Table S3: Renal function at baseline of population-based and disease-cohorts included**

| Cohort | Country | N | Creatinine (mg/dL)* | Cystatin C (mg/dL)* | eGFR CKD-EPI crea (ml/min/1.73m²)* | CKDcrea stage 3+ (%) | eGFR CKD-EPI cysC (ml/min/1.73m²)* | CKDcysC stage 3+ (%) |
| --- | --- | --- | --- | --- | --- | --- | --- | --- |
| **Population-based cohorts** n=20 |  |  |  |  |  |  |  |  |
| MONICA/KORA S3 | Germany | 4640 | 0.74 (0.64,0.85) | 0.77 (0.69,0.86) | 102.1 (91.4,112.8) | 103 (2.2%) | 101.2 (86.6,116.2) | 127 (2.7%) |
| MONICA/KORA S4 | Germany | 3558 | 0.82 (0.73,0.94) | 0.84 (0.75,0.94) | 95.6 (83.2,106.7) | 82 (2.3%) | 92.0 (78.4,105.2) | 177 (5.0%) |
| Catalonia 86 | Spain | 2392 | 0.71 (0.63,0.80) | 0.73 (0.63,0.83) | 106.6 (97.6,115.7) | 16 (0.7%) | 107.9 (91.7,127.6) | 40 (1.7%) |
| Catalonia 90 | Spain | 2731 | 0.66 (0.58,0.75) | 0.69 (0.58,0.80) | 112.9 (104.2,122.2) | 7 (0.3%) | 116.5 (97.6,141.5) | 37 (1.4%) |
| FINRISK 97 | Finland | 4591 | 0.90 (0.80,1.00) | 0.80 (0.70,0.90) | 90.6 (78.6,102.0) | 230 (5.0%) | 95.3 (82.7,108.2) | 153 (3.3%) |
| DAN-MONICA 1 | Denmark | 3708 | 0.80 (0.70,0.92) | 0.71 (0.63,0.81) | 101.1 (88.9,111.2) | 105 (2.8%) | 111.7 (95.6,129.3) | 76 (2.0%) |
| DAN-MONICA 2 | Denmark | 1494 | 0.81 (0.71,0.93) | 0.71 (0.62,0.81) | 98.5 (88.3,110.0) | 20 (1.3%) | 111.1 (95.5,131.3) | 19 (1.3%) |
| DAN-MONICA 3 | Denmark | 2005 | 0.83 (0.72,0.95) | 0.72 (0.64,0.83) | 94.9 (82.4,108.2) | 76 (3.8%) | 108.2 (90.7,126.4) | 45 (2.2%) |
| Moli-sani | Italy | 23646 | 0.78 (0.70,0.88) | 0.95 (0.84,1.08) | 94.8 (84.3,103.5) | 825 (3.5%) | 77.7 (65.8,89.8) | 3781 (16.0%) |
| N-Sweden 86 | Sweden | 1601 | 0.70 (0.62,0.79) | 0.79 (0.69,0.90) | 108.5 (100.3,116.7) | 8 (0.5%) | 99.5 (84.3,116.0) | 44 (2.7%) |
| N-Sweden 90 | Sweden | 1420 | 0.71 (0.64,0.79) | 0.85 (0.75,0.95) | 107.7 (98.9,116.7) | 7 (0.5%) | 91.2 (78.4,105.0) | 67 (4.7%) |
| N-Sweden 94 | Sweden | 1853 | 0.78 (0.70,0.89) | 0.94 (0.81,1.08) | 97.8 (86.3,109.2) | 44 (2.4%) | 80.5 (65.9,95.5) | 267 (14.4%) |
| N-Sweden 99 | Sweden | 1725 | 0.79 (0.69,0.90) | 0.91 (0.78,1.07) | 96.7 (83.6,108.9) | 73 (4.2%) | 82.4 (66.8,99.9) | 248 (14.4%) |
| N-Sweden 4 | Sweden | 1810 | 0.64 (0.55,0.74) | 0.70 (0.59,0.82) | 108.5 (97.6,119.0) | 29 (1.6%) | 112.8 (92.1,139.0) | 80 (4.4%) |
| N-Sweden 9 | Sweden | 1627 | 0.71 (0.62,0.82) | 0.83 (0.71,0.99) | 102.3 (91.1,113.5) | 58 (3.6%) | 92.1 (74.6,110.9) | 149 (9.2%) |
| PRIME Belfast | UK | 1856 | 1.00 (0.90,1.10) | 1.00 (0.90,1.10) | 85.0 (73.6,97.2) | 201 (10.8%) | 76.6 (68.5,86.6) | 90 (4.8%) |
| SHHEC 1 | UK | 10739 | 0.80 (0.73,0.94) | 0.70 (0.65,0.80) | 96.4 (84.3,104.4) | 443 (4.1%) | 106.4 (92.0,124.3) | 74 (0.7%) |
| SHHEC 21 | UK | 680 | 0.79 (0.72,0.91) | 0.70 (0.70,0.80) | 97.7 (86.3,106.6) | 9 (1.3%) | 107.2 (91.3,125.6) | 6 (0.9%) |
| SHHEC 2 | UK | 1651 | 0.79 (0.72,0.90) | 0.80 (0.70,0.90) | 95.3 (83.2,107.1) | 83 (5.0%) | 100.6 (84.0,119.6) | 63 (3.8%) |
| SHHEC 3 | UK | 1640 | 0.82 (0.75,0.93) | 0.69 (0.63,0.77) | 97.0 (86.2,106.8) | 31 (1.9%) | 114.8 (99.6,129.6) | 19 (1.2%) |
| Total |  | 75367 | 0.79 (0.70,0.90) | 0.81 (0.70,0.96) | 97.6 (85.9,107.6) | 2450 (3.3%) | 92.5 (76.4,111.5) | 5562 (7.4%) |
| **Disease cohorts** n=3 |  |  |  |  |  |  |  |  |
| AtheroGene | Germany | 2137 | 0.94 (0.83,1.08) | 0.82 (0.71,0.97) | 82.3 (68.3,93.8) | 303 (14.2%) | 92.9 (75.5,110.2) | 211 (9.9%) |
| KAROLA | Germany | 1129 | 0.90 (0.79,1.00) | 1.11 (1.01,1.28) | 89.3 (75.9,98.1) | 71 (6.3%) | 65.3 (54.9,74.4) | 404 (35.8%) |
| StenoCardia | Germany | 1716 | 0.95 (0.83,1.09) | 0.67 (0.58,0.80) | 80.0 (65.1,92.0) | 317 (18.5%) | 116.9 (92.9,141.0) | 104 (6.1%) |
| Total |  | 4982 | 0.93 (0.82,1.07) | 0.83 (0.68,1.05) | 82.7 (68.7,94.6) | 691 (13.9%) | 91.1 (69.1,116.2) | 719 (14.4%) |

* Values indicate median and the first and third quartile (Q1, Q3).

**Figure S1: Distribution of the estimated glomerular filtration rate (eGFR) based on creatinine and cystatin C in the population based cohorts**


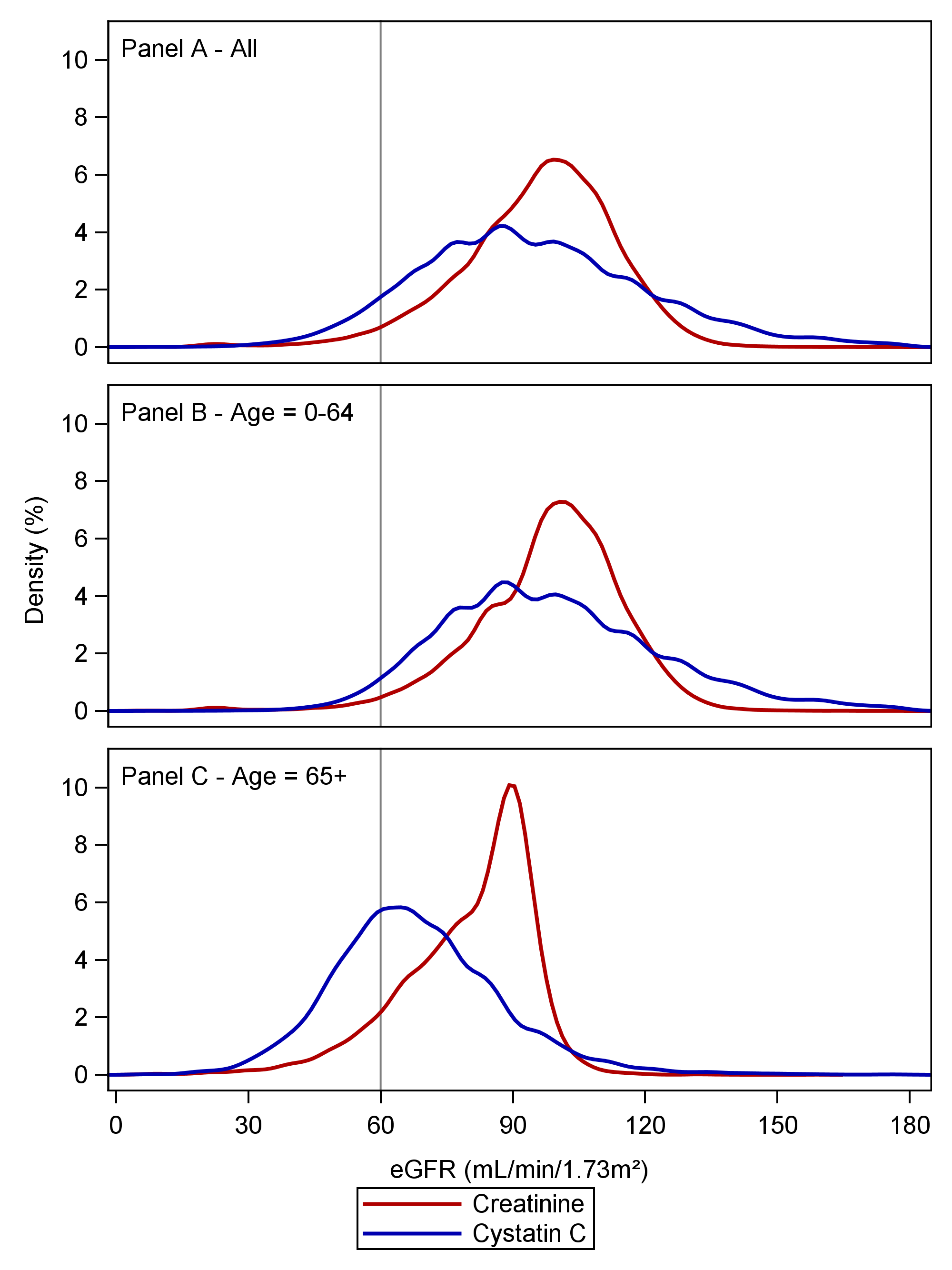


**Figure S2: Distribution of the estimated glomerular filtration rate (eGFR) based on creatinine and cystatin C in the disease cohorts**


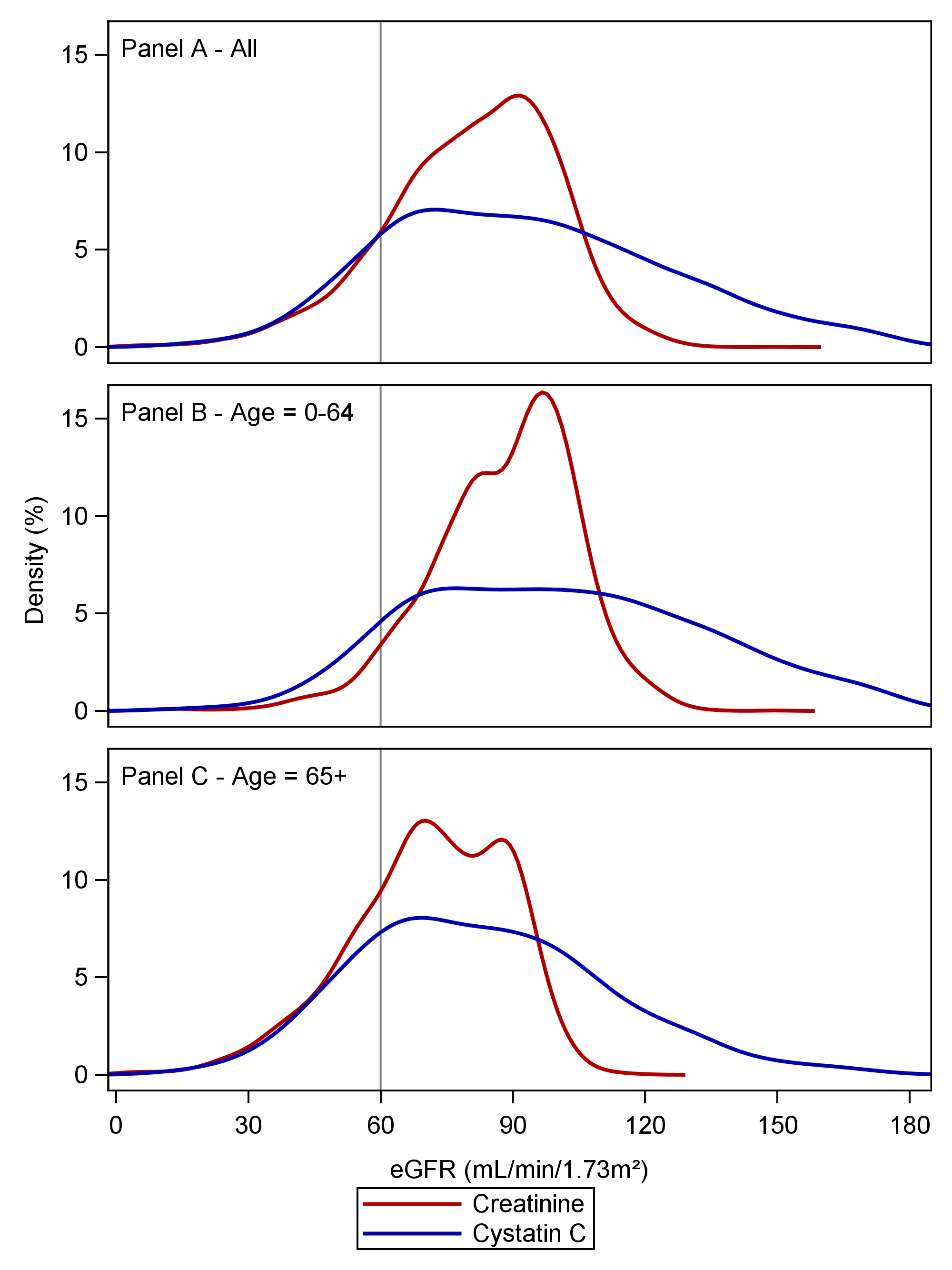


**Table S4: Association of CKD with various endpoints (HR and 95% CI) including area-under the curve (AUC with 95% CI) and net-reclassification index (NRI with 95% CI) in population-based cohorts**

| **Panel A: Cardiovascular Mortality** | |  |  |  |  |
| --- | --- | --- | --- | --- | --- |
|  |  | **HR (95% CI)** | **AUC (95% CI)^a^** | **Event NRI (95% CI)** | **Non-event NRI (95% CI)** |
| **All** | CKDcrea | 1.72 (1.53-1.92) | 0.84 (0.83-0.85) | -0.9% (-2.1%,0.3%) | 0.7% (0.6%,0.8%) |
|  | CKDcys | 2.14 (1.90-2.40) | 0.84 (0.84-0.85) | -0.7% (-2.4%,0.9%) | 2.0% (1.8%,2.2%) |
| **Age < 65** | CKDcrea | 1.53 (1.32-1.79) | 0.83 (0.82-0.83) | 0.9% (-0.3%,2.0%) | 0.1% (0.0%,0.2%) |
|  | CKDcys | 2.36 (1.99-2.78) | 0.83 (0.82-0.84) | 2.8% (1.0%,4.5%) | 0.5% (0.3%,0.6%) |
| **Age >= 65** | CKDcrea | 1.92 (1.61-2.29) | 0.73 (0.71-0.74) | -1.0% (-2.7%,0.7%) | 3.1% (2.5%,3.8%) |
|  | CKDcys | 1.93 (1.64-2.28) | 0.73 (0.71-0.75) | -1.0% (-3.2%,1.2%) | 4.2% (3.2%,5.1%) |
| **Male Sex** | CKDcrea | 1.68 (1.45-1.96) | 0.81 (0.81-0.82) | -0.6% (-1.8%,0.6%) | 0.9% (0.8%,1.1%) |
|  | CKDcys | 2.01 (1.72-2.34) | 0.81 (0.81-0.82) | -3.0% (-4.8%,-1.3%) | 2.5% (2.2%,2.8%) |
| **Female Sex** | CKDcrea | 1.77 (1.48-2.12) | 0.86 (0.85-0.87) | 1.7% (-0.8%,4.2%) | 0.7% (0.5%,0.8%) |
|  | CKDcys | 2.33 (1.94-2.80) | 0.86 (0.85-0.87) | 3.7% (-0.2%,7.6%) | 1.5% (1.3%,1.8%) |
| **No Hypertension** | CKDcrea | 1.63 (1.31-2.03) | 0.85 (0.84-0.86) | 1.4% (-0.1%,2.9%) | 0.1% (0.0%,0.2%) |
|  | CKDcys | 2.62 (2.11-3.26) | 0.85 (0.84-0.86) | 1.5% (-1.0%,4.1%) | 0.8% (0.6%,0.9%) |
| **Hypertension** | CKDcrea | 1.72 (1.50-1.97) | 0.78 (0.77-0.79) | -1.1% (-2.4%,0.2%) | 1.2% (1.0%,1.5%) |
|  | CKDcys | 2.01 (1.76-2.31) | 0.78 (0.77-0.79) | -0.4% (-2.3%,1.6%) | 3.8% (3.4%,4.2%) |
| **No Diabetes** | CKDcrea | 1.65 (1.45-1.87) | 0.84 (0.83-0.85) | 0.1% (-1.1%,1.3%) | 0.6% (0.5%,0.7%) |
|  | CKDcys | 2.04 (1.80-2.32) | 0.84 (0.83-0.85) | -0.1% (-1.7%,1.6%) | 1.7% (1.5%,1.8%) |
| **Diabetes** | CKDcrea | 1.72 (1.29-2.31) | 0.78 (0.76-0.80) | -1.3% (-3.9%,1.3%) | 2.5% (1.5%,3.5%) |
|  | CKDcys | 2.50 (1.87-3.33) | 0.78 (0.76-0.81) | -2.6% (-6.3%,1.0%) | 6.6% (4.9%,8.3%) |
| ^a^AUC (95% CI) for model without CKD 0.84 (0.83-0.84) | | |  |  |  |
| **Panel B: Cardiovascular Disease** | |  |  |  |  |
|  |  | **HR (95% CI)** | **AUC (95% CI)** | **Event NRI (95% CI)** | **Non-event NRI (95% CI)** |
| **All** | CKDcrea | 1.27 (1.15-1.41) | 0.79 (0.78-0.79) | 0.0% (-0.3%,0.3%) | 0.3% (0.2%,0.3%) |
|  | CKDcys | 1.40 (1.26-1.55) | 0.79 (0.78-0.79) | 0.3% (-0.3%,0.8%) | 1.2% (1.1%,1.4%) |
| **Age < 65** | CKDcrea | 1.25 (1.10-1.42) | 0.78 (0.77-0.78) | -0.1% (-0.4%,0.3%) | 0.0% (-0.1%,0.0%) |
|  | CKDcys | 1.61 (1.40-1.85) | 0.78 (0.77-0.78) | -0.4% (-1.2%,0.3%) | 0.5% (0.3%,0.6%) |
| **Age >= 65** | CKDcrea | 1.36 (1.13-1.64) | 0.67 (0.65-0.69) | -1.1% (-1.7%,-0.5%) | 0.8% (0.4%,1.3%) |
|  | CKDcys | 1.28 (1.09-1.49) | 0.67 (0.65-0.68) | -1.4% (-2.2%,-0.6%) | 0.8% (0.1%,1.5%) |
| **Male Sex** | CKDcrea | 1.23 (1.07-1.41) | 0.75 (0.75-0.76) | 0.0% (-0.4%,0.3%) | 0.2% (0.0%,0.3%) |
|  | CKDcys | 1.34 (1.17-1.53) | 0.75 (0.75-0.76) | -0.8% (-1.4%,-0.2%) | 1.1% (0.9%,1.2%) |
| **Female Sex** | CKDcrea | 1.33 (1.13-1.56) | 0.80 (0.79-0.81) | -0.7% (-1.7%,0.4%) | 0.1% (0.0%,0.3%) |
|  | CKDcys | 1.52 (1.30-1.78) | 0.80 (0.79-0.81) | -2.4% (-4.1%,-0.7%) | 1.3% (1.1%,1.5%) |
| **No Hypertension** | CKDcrea | 1.17 (0.97-1.41) | 0.79 (0.78-0.80) | -0.2% (-0.8%,0.4%) | -0.1% (-0.1%,0.0%) |
|  | CKDcys | 1.69 (1.39-2.05) | 0.79 (0.78-0.80) | -0.6% (-1.8%,0.7%) | 0.5% (0.4%,0.7%) |
| **Hypertension** | CKDcrea | 1.32 (1.16-1.49) | 0.72 (0.71-0.73) | -0.3% (-0.7%,0.1%) | 0.7% (0.5%,0.9%) |
|  | CKDcys | 1.29 (1.15-1.46) | 0.72 (0.71-0.73) | -0.2% (-0.8%,0.4%) | 1.1% (0.8%,1.4%) |
| **No Diabetes** | CKDcrea | 1.23 (1.10-1.37) | 0.78 (0.78-0.79) | -0.5% (-0.9%,-0.1%) | 0.2% (0.1%,0.2%) |
|  | CKDcys | 1.35 (1.20-1.51) | 0.78 (0.78-0.79) | -0.7% (-1.3%,0.0%) | 1.0% (0.9%,1.2%) |
| **Diabetes** | CKDcrea | 1.31 (0.97-1.75) | 0.72 (0.70-0.74) | -0.4% (-1.0%,0.1%) | 0.8% (0.3%,1.3%) |
|  | CKDcys | 1.57 (1.20-2.06) | 0.72 (0.70-0.75) | -1.4% (-2.8%,0.1%) | 2.8% (1.7%,3.8%) |
| ^a^AUC (95% CI) for model without CKD 0.78 (0.78-0.84) | | |  |  |  |
| **Panel C: Total Mortality** | |  |  |  |  |
|  |  | **HR (95% CI)** | **AUC (95% CI)** | **Event NRI (95% CI)** | **Non-event NRI (95% CI)** |
| **All** | CKDcrea | 1.38 (1.27-1.49) | 0.79 (0.79-0.80) | 0.2% (-0.1%,0.6%) | 0.5% (0.4%,0.6%) |
|  | CKDcys | 1.88 (1.74-2.03) | 0.80 (0.79-0.80) | -1.9% (-2.6%,-1.2%) | 4.1% (3.9%,4.3%) |
| **Age < 65** | CKDcrea | 1.29 (1.16-1.43) | 0.77 (0.77-0.78) | 0.4% (0.0%,0.8%) | 0.1% (0.0%,0.2%) |
|  | CKDcys | 2.10 (1.89-2.34) | 0.77 (0.77-0.78) | 1.0% (0.1%,2.0%) | 1.4% (1.2%,1.6%) |
| **Age >= 65** | CKDcrea | 1.46 (1.29-1.66) | 0.69 (0.68-0.70) | 0.0% (-0.2%,0.2%) | 1.1% (0.8%,1.5%) |
|  | CKDcys | 1.72 (1.54-1.91) | 0.70 (0.68-0.71) | 0.1% (-0.6%,0.7%) | 6.5% (5.7%,7.2%) |
| **Male Sex** | CKDcrea | 1.35 (1.21-1.50) | 0.78 (0.77-0.78) | -0.1% (-0.4%,0.3%) | 0.2% (0.1%,0.4%) |
|  | CKDcys | 1.83 (1.66-2.02) | 0.78 (0.77-0.79) | -1.8% (-2.4%,-1.1%) | 3.9% (3.7%,4.2%) |
| **Female Sex** | CKDcrea | 1.41 (1.25-1.59) | 0.80 (0.79-0.81) | 0.1% (-0.7%,0.8%) | 0.5% (0.4%,0.7%) |
|  | CKDcys | 1.99 (1.77-2.24) | 0.80 (0.80-0.81) | -2.3% (-3.9%,-0.6%) | 3.9% (3.6%,4.2%) |
| **No Hypertension** | CKDcrea | 1.35 (1.17-1.55) | 0.80 (0.79-0.80) | 0.2% (-0.3%,0.8%) | 0.1% (0.0%,0.2%) |
|  | CKDcys | 2.06 (1.81-2.36) | 0.80 (0.79-0.80) | -1.5% (-2.6%,-0.4%) | 2.3% (2.1%,2.5%) |
| **Hypertension** | CKDcrea | 1.39 (1.26-1.53) | 0.75 (0.75-0.76) | 0.1% (-0.3%,0.5%) | 0.7% (0.5%,0.9%) |
|  | CKDcys | 1.87 (1.71-2.05) | 0.76 (0.75-0.77) | -1.4% (-2.3%,-0.5%) | 5.7% (5.3%,6.1%) |
| **No Diabetes** | CKDcrea | 1.31 (1.20-1.42) | 0.79 (0.79-0.80) | -0.4% (-0.7%,0.0%) | 0.3% (0.2%,0.4%) |
|  | CKDcys | 1.80 (1.66-1.95) | 0.80 (0.79-0.80) | -1.5% (-2.2%,-0.8%) | 3.4% (3.2%,3.6%) |
| **Diabetes** | CKDcrea | 1.64 (1.33-2.02) | 0.73 (0.71-0.75) | 0.3% (-0.2%,0.8%) | 2.5% (1.7%,3.3%) |
|  | CKDcys | 2.26 (1.85-2.77) | 0.74 (0.72-0.76) | -1.7% (-3.3%,-0.1%) | 9.4% (8.0%,10.9%) |

^a^AUC (95% CI) for model without CKD 0.79 (0.79-0.80)

Models adjusted for age, sex, cohort, smoking status, systolic blood pressure and total cholesterol.

**Table S5: Association of CKD with various endpoints (HR and 95% CI) including area-under the curve (AUC with 95% CI) and net-reclassification index (NRI with 95% CI) in disease cohorts**

| **Panel A: Cardiovascular Mortality** | |  |  |  |  |
| --- | --- | --- | --- | --- | --- |
|  |  | **HR (95% CI)** | **AUC (95% CI)** | **Event NRI (95% CI)** | **Non-event NRI (95% CI)** |
| **All** | CKDcrea | 3.33 (2.14-5.19) | 0.77 (0.73-0.82) | -5.9% (-13.6%,1.8%) | 5.0% (3.5%,6.4%) |
|  | CKDcys | 3.20 (2.04-5.01) | 0.77 (0.73-0.82) | 3.5% (-3.4%,10.4%) | -0.2% (-1.5%,1.2%) |
| **Age < 65** | CKDcrea | 6.91 (3.48-13.70) | 0.76 (0.68-0.83) | 3.8% (-5.9%,13.4%) | 6.3% (4.9%,7.7%) |
|  | CKDcys | 4.96 (2.38-10.37) | 0.76 (0.69-0.83) | 7.7% (-2.4%,17.8%) | 4.6% (2.9%,6.3%) |
| **Age >= 65** | CKDcrea | 2.27 (1.30-3.94) | 0.76 (0.71-0.81) | 1.7% (-4.7%,8.1%) | 4.1% (2.5%,5.7%) |
|  | CKDcys | 2.59 (1.46-4.59) | 0.77 (0.72-0.82) | -1.2% (-5.2%,2.8%) | -5.7% (-7.2%,-4.1%) |
| **Male Sex** | CKDcrea | 3.42 (2.07-5.66) | 0.76 (0.71-0.81) | -3.3% (-11.8%,5.1%) | 6.4% (4.8%,7.9%) |
|  | CKDcys | 2.49 (1.49-4.16) | 0.74 (0.69-0.80) | 3.7% (-3.0%,10.4%) | -3.7% (-5.0%,-2.3%) |
| **Female Sex** | CKDcrea | 2.59 (1.00-6.72) | 0.83 (0.72-0.94) | 12.8% (0.6%,24.9%) | 6.3% (3.5%,9.1%) |
|  | CKDcys | 7.76 (2.70-22.32) | 0.87 (0.78-0.96) | -0.1% (-15.9%,15.8%) | 15.8% (12.2%,19.5%) |
| **No Hypertension** | CKDcrea | 6.20 (2.19-17.53) | 0.78 (0.70-0.86) | -1.0% (-16.6%,14.6%) | 6.4% (4.3%,8.5%) |
|  | CKDcys | 2.31 (0.84-6.32) | 0.77 (0.68-0.85) | -5.0% (-17.1%,7.1%) | 1.7% (0.2%,3.3%) |
| **Hypertension** | CKDcrea | 2.88 (1.75-4.71) | 0.77 (0.72-0.82) | -1.7% (-9.8%,6.4%) | 5.6% (3.8%,7.4%) |
|  | CKDcys | 3.48 (2.09-5.79) | 0.78 (0.73-0.83) | 1.4% (-6.0%,8.9%) | 2.9% (1.2%,4.6%) |
| **No Diabetes** | CKDcrea | 3.58 (2.06-6.24) | 0.77 (0.72-0.83) | 9.1% (-0.1%,18.3%) | 4.5% (2.9%,6.0%) |
|  | CKDcys | 2.87 (1.60-5.18) | 0.77 (0.71-0.83) | -0.3% (-5.1%,4.4%) | 0.3% (-1.1%,1.7%) |
| **Diabetes** | CKDcrea | 2.74 (1.31-5.70) | 0.73 (0.64-0.81) | 10.3% (2.3%,18.3%) | 6.8% (3.5%,10.1%) |
|  | CKDcys | 3.32 (1.64-6.73) | 0.75 (0.68-0.82) | 10.4% (-3.0%,23.8%) | 5.6% (1.7%,9.6%) |
| ^a^AUC (95% CI) for model without CKD 0.74 (0.69-0.79) | | |  |  |  |
| **Panel B: Cardiovascular Disease** | |  |  |  |  |
|  |  | **HR (95% CI)** | **AUC (95% CI)** | **Event NRI (95% CI)** | **Non-event NRI (95% CI)** |
| **All** | CKDcrea | 2.03 (1.52-2.71) | 0.62 (0.59-0.66) | 0.5% (-0.1%,1.2%) | 0.2% (-0.4%,0.8%) |
|  | CKDcys | 2.23 (1.71-2.90) | 0.64 (0.60-0.67) | 0.1% (-1.8%,1.9%) | 2.1% (1.1%,3.1%) |
| **Age < 65** | CKDcrea | 2.73 (1.70-4.36) | 0.60 (0.56-0.65) | 0.6% (-2.1%,3.4%) | 5.5% (4.4%,6.6%) |
|  | CKDcys | 2.53 (1.72-3.74) | 0.64 (0.59-0.68) | -2.5% (-6.8%,1.7%) | 8.9% (7.2%,10.7%) |
| **Age >= 65** | CKDcrea | 1.70 (1.17-2.46) | 0.63 (0.58-0.67) | 0.0% (0.0%,0.0%) | 0.0% (0.0%,0.0%) |
|  | CKDcys | 1.96 (1.37-2.82) | 0.63 (0.58-0.68) | 0.0% (0.0%,0.0%) | 0.3% (0.0%,0.6%) |
| **Male Sex** | CKDcrea | 1.95 (1.37-2.76) | 0.61 (0.57-0.65) | 0.4% (-0.1%,0.8%) | -1.1% (-1.6%,-0.6%) |
|  | CKDcys | 2.12 (1.57-2.86) | 0.62 (0.59-0.66) | -2.4% (-4.1%,-0.7%) | 1.3% (0.4%,2.3%) |
| **Female Sex** | CKDcrea | 2.16 (1.27-3.69) | 0.68 (0.61-0.74) | 10.1% (4.8%,15.3%) | 11.8% (9.2%,14.5%) |
|  | CKDcys | 2.73 (1.54-4.83) | 0.67 (0.60-0.75) | 3.6% (-1.5%,8.8%) | 10.2% (7.6%,12.8%) |
| **No Hypertension** | CKDcrea | 2.23 (1.07-4.63) | 0.61 (0.55-0.68) | -1.0% (-4.4%,2.3%) | 2.0% (0.6%,3.4%) |
|  | CKDcys | 2.26 (1.36-3.76) | 0.63 (0.57-0.69) | -1.1% (-7.5%,5.4%) | 10.8% (8.2%,13.3%) |
| **Hypertension** | CKDcrea | 1.92 (1.40-2.65) | 0.63 (0.59-0.67) | 0.3% (-0.2%,0.8%) | -1.0% (-1.5%,-0.4%) |
|  | CKDcys | 2.18 (1.59-2.98) | 0.64 (0.60-0.68) | -0.7% (-1.8%,0.5%) | 1.5% (0.6%,2.5%) |
| **No Diabetes** | CKDcrea | 1.77 (1.21-2.58) | 0.61 (0.57-0.65) | 0.1% (-1.1%,1.3%) | 1.3% (0.6%,2.0%) |
|  | CKDcys | 1.87 (1.34-2.61) | 0.61 (0.57-0.65) | -3.0% (-4.9%,-1.2%) | 2.1% (1.0%,3.2%) |
| **Diabetes** | CKDcrea | 2.42 (1.51-3.88) | 0.62 (0.57-0.68) | 0.0% (0.0%,0.0%) | 0.8% (0.0%,1.7%) |
|  | CKDcys | 2.64 (1.70-4.11) | 0.66 (0.61-0.71) | 0.0% (0.0%,0.0%) | 0.2% (-0.6%,0.9%) |
| ^a^AUC (95% CI) for model without CKD 0.60 (0.57-0.64) | | |  |  |  |
| **Panel C: Total Mortality** | |  |  |  |  |
|  |  | **HR (95% CI)** | **AUC (95% CI)** | **Event NRI (95% CI)** | **Non-event NRI (95% CI)** |
| **All** | CKDcrea | 2.60 (1.96-3.46) | 0.73 (0.70-0.76) | -0.5% (-1.9%,0.9%) | 1.7% (0.8%,2.5%) |
|  | CKDcys | 2.32 (1.78-3.02) | 0.73 (0.70-0.76) | -0.8% (-2.4%,0.8%) | -0.4% (-1.6%,0.8%) |
| **Age < 65** | CKDcrea | 5.15 (3.31-8.03) | 0.71 (0.66-0.77) | -3.5% (-7.2%,0.2%) | 11.4% (9.8%,13.1%) |
|  | CKDcys | 2.37 (1.58-3.57) | 0.69 (0.64-0.74) | -2.9% (-6.6%,0.8%) | 4.5% (2.8%,6.2%) |
| **Age >= 65** | CKDcrea | 1.79 (1.25-2.57) | 0.67 (0.63-0.72) | 0.0% (0.0%,0.0%) | 0.1% (-0.1%,0.2%) |
|  | CKDcys | 2.33 (1.65-3.28) | 0.69 (0.64-0.73) | -0.2% (-0.7%,0.2%) | 1.2% (0.6%,1.8%) |
| **Male Sex** | CKDcrea | 2.55 (1.83-3.54) | 0.73 (0.70-0.76) | 1.1% (0.2%,2.0%) | -0.7% (-1.4%,0.0%) |
|  | CKDcys | 2.06 (1.54-2.75) | 0.72 (0.69-0.75) | 0.1% (-1.5%,1.7%) | -0.8% (-2.0%,0.4%) |
| **Female Sex** | CKDcrea | 2.65 (1.48-4.77) | 0.73 (0.66-0.81) | -3.9% (-11.7%,4.0%) | 11.6% (8.6%,14.7%) |
|  | CKDcys | 3.96 (2.09-7.52) | 0.77 (0.71-0.83) | 1.7% (-3.3%,6.8%) | 5.6% (2.2%,8.9%) |
| **No Hypertension** | CKDcrea | 3.05 (1.65-5.64) | 0.74 (0.68-0.79) | 0.0% (0.0%,0.0%) | 2.7% (1.1%,4.3%) |
|  | CKDcys | 2.09 (1.30-3.37) | 0.72 (0.67-0.78) | -2.3% (-4.5%,-0.1%) | -0.6% (-3.0%,1.7%) |
| **Hypertension** | CKDcrea | 2.50 (1.81-3.46) | 0.72 (0.69-0.76) | 0.8% (-0.5%,2.2%) | 1.1% (0.1%,2.0%) |
|  | CKDcys | 2.43 (1.77-3.34) | 0.72 (0.69-0.76) | -0.9% (-2.5%,0.6%) | -0.1% (-1.4%,1.2%) |
| **No Diabetes** | CKDcrea | 2.30 (1.59-3.33) | 0.74 (0.70-0.78) | -4.5% (-6.4%,-2.6%) | 1.0% (0.2%,1.9%) |
|  | CKDcys | 2.20 (1.57-3.07) | 0.74 (0.70-0.78) | -0.4% (-2.6%,1.8%) | -1.4% (-2.7%,-0.1%) |
| **Diabetes** | CKDcrea | 3.09 (1.95-4.90) | 0.68 (0.62-0.73) | 0.5% (-0.4%,1.3%) | 0.2% (-0.8%,1.2%) |
|  | CKDcys | 2.25 (1.47-3.43) | 0.67 (0.62-0.72) | 1.0% (-0.3%,2.3%) | 0.1% (-0.9%,1.0%) |

^a^AUC (95% CI) for model without CKD 0.70 (0.67-0.74)

Models adjusted for age, sex, cohort, smoking status, systolic blood pressure and total cholesterol.

**Table S6: Agreement of reclassification by the CKD-EPIcysC based definition compared to**

**CKD-EPIcrea based definition in the population-based cohorts (Panel A) and in the disease cohorts (Panel B)**

| **Panel A** |  | CKD-cysC |  |  | Reclassification by CKDcysC (%) | |
| --- | --- | --- | --- | --- | --- | --- |
|  |  | No | Yes |  | Up | Down |
| CKDcrea (n, %) | No | 68643 | 4274 |  |  | 5.9% |
|  |  | 94.1% | 5.9% |  |  |  |
|  | Yes | 1162 | 1288 |  | 47.4% |  |
|  |  | 47.4% | 52.6% |  |  |  |
|  |  |  |  |  |  |  |
| **Panel B** |  | CKD-cysC |  |  | Reclassification by CKDcysC (%) | |
|  |  | No | Yes |  | Up | Down |
| CKDcrea (n, %) | No | 3879 | 412 |  |  | 9.6% |
|  |  | 90.4% | 9.6% |  |  |  |
|  | Yes | 384 | 307 |  | 55.6% |  |
|  |  | 55.6% | 44.4% |  |  |  |

CKD was defined as eGFR < 60mL/min/1.72m^2^.

Table shows agreement when using both CKD-EPI definitions and reclassification when CKD-EPIcysC is used
compared to CKD-EPIcrea

**Figure S7: Spline regression representing creatinine (left side) and cystatin C (right side) based eGFR associated with hazard ratio`s (HR) for mortality after adjustment for SCORE-variables and study center for all and after stratification for age in the disease cohorts**


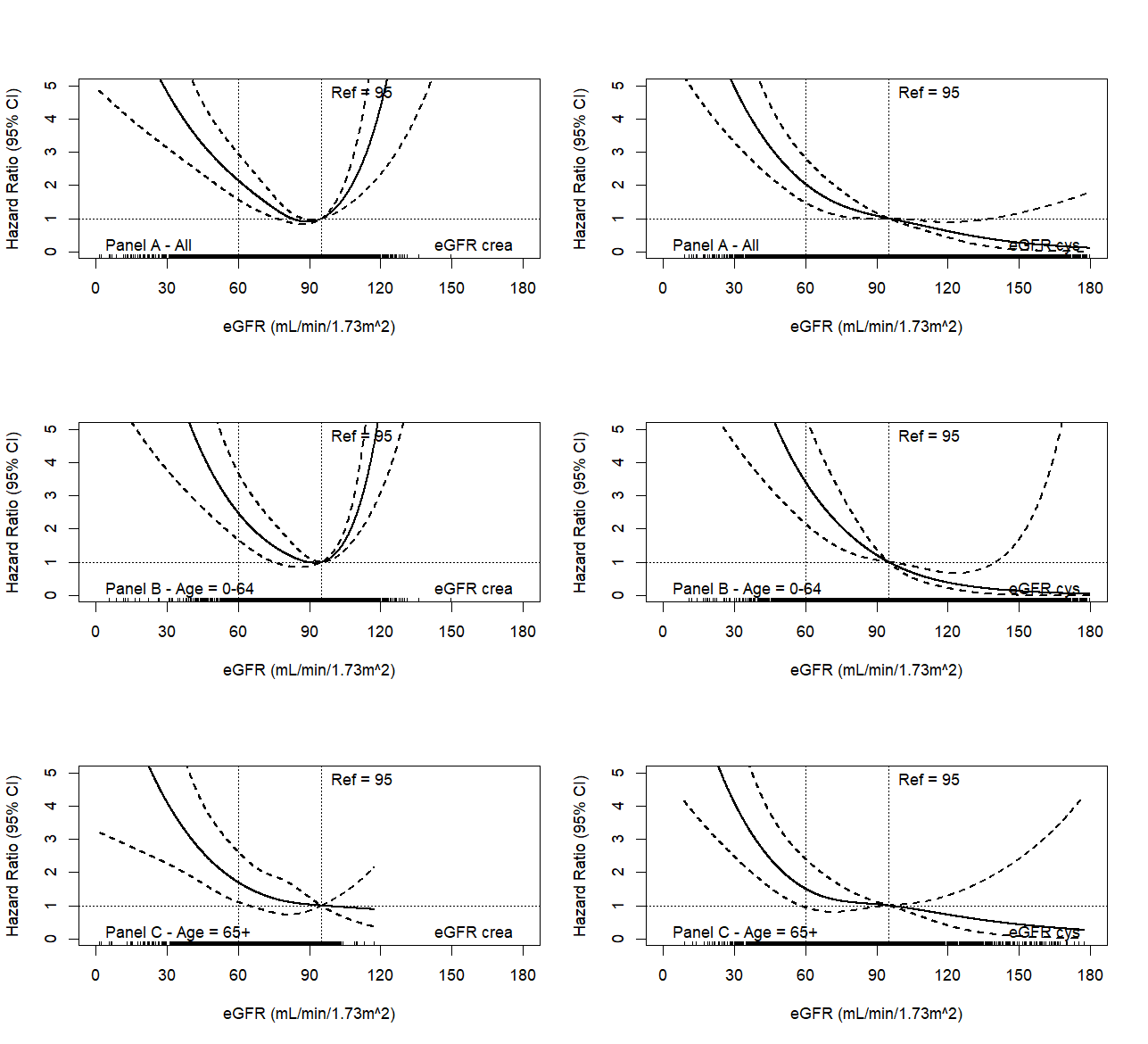


**Figure S8: Area under the curve (AUC) for creatinine and cystatin C based eGFR
in population-based (left panels) and disease cohorts (right panels) for all-cause**


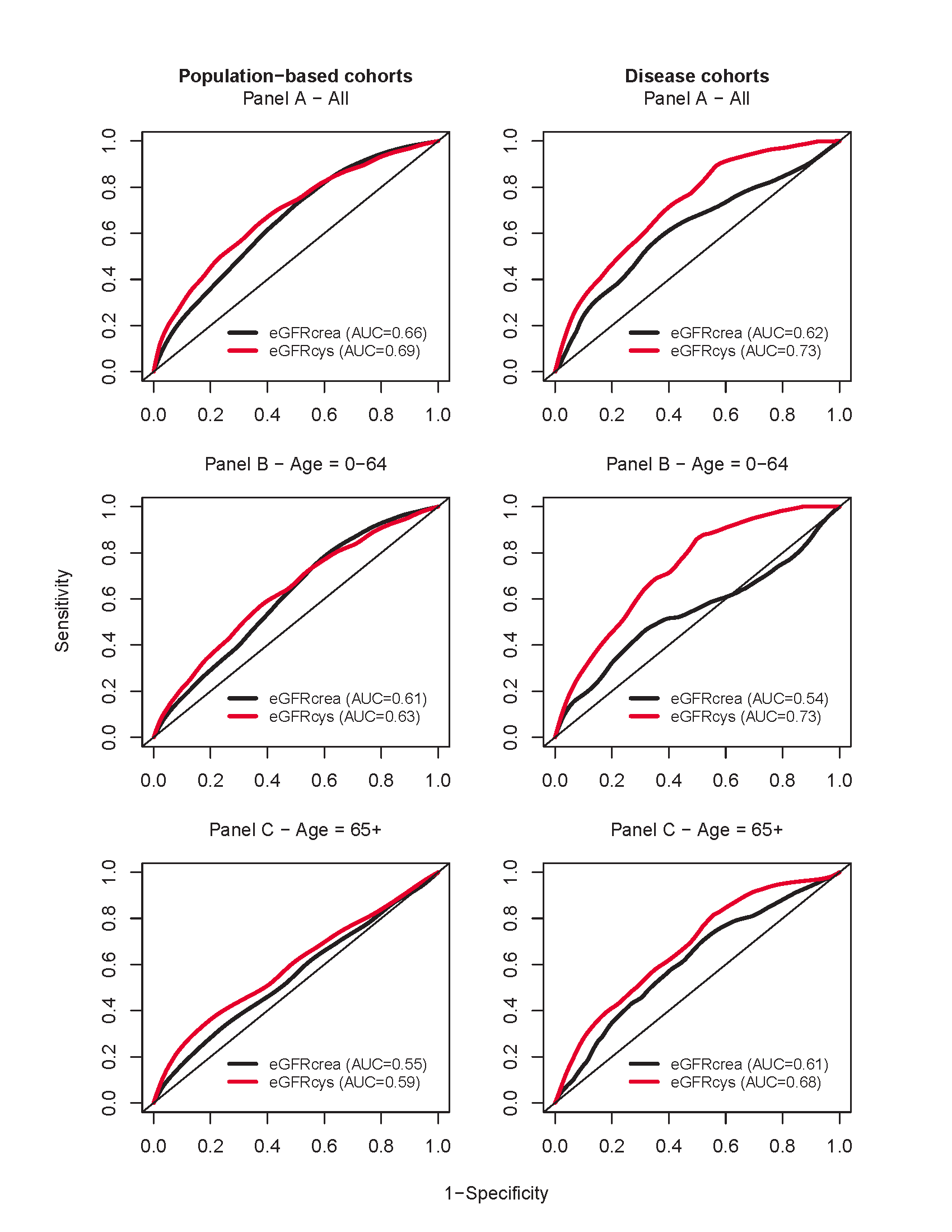


**Box S1: Further Description of Study Cohorts**

| **General Population Based Cohorts** |
| --- |
| **Kooperative Gesundheitsforschung in der Region Augsburg (MONICA/KORA)**: The WHO Multinational Monitoring of Trends and Determinants in Cardiovascular Diseases (MONICA)/ Cooperative Health Research in the Region of Augsburg (KORA) cohorts were randomly selected from representative sample surveys from the city of Augsburg and the less urban Landkreis Augsburg and Landkreis Aichach-Friedberg regions in Bavaria, Southern Germany. List of municipalities and population registers were used as sampling frames for the first and the second stage of two-stage sampling, respectively. The second stage of sampling was stratified by sex and 10-year age group. The Survey 3 (S3) baseline examination (1994-1995) was carried out as part of the WHO MONICA project and consists of 4,480 men and women aged 25-74 with a response rate of 74%. ^12^ The Survey 4 baseline examination (S4) was carried out in 1999-2001 consisting of 3,019 men and women aged 25-74 who were re-examined with repeated measurements collected, in 2006-2008 (F4) with a response rate of 72%.^13^ S4 and F4 studies and morbidity and mortality follow-ups were conducted in the frame of KORA. Coronary events were identified through the MONICA/KORA Augsburg coronary event registry. Coronary deaths were validated by autopsy reports, death certificates, chart review from the last treating physician. Cases with self-reported incident diabetes were validated by the treating physician and medical records documenting use of antidiabetic medication. Self-reported cases of incident stroke were validated by medical records. Mortality follow-up until 2009 was conducted through national death registers. <http://www.thl.fi/publications/morgam/cohorts/full/germany/ger-auga.htm> |
| **MONICA-Catalonia Study:** The Catalonia Study consists of two cohorts sampled from representative surveys from the central area of Catalonia and part of the metropolitan area of Barcelona, Spain. The first stage drew a random sample of individuals from nine municipalities with probability proportional to population size. In the second stage, a random sample of men and women from the Municipal population registries were used as the sampling frame, from each municipal population register, a random sample stratified by sex and ten-year age groups, 25-64 years, of fixed number was selected. Baseline examinations were carried out in 1986-1988 for cohort 1 of N=2,571, response rate was 74%. Cohort 2 was collected in 1990-1992 (N=2,936), response rate was 67%. Participants were mainly from the Industrial and services economy.^14-16^ All subjects gave informed consent. The project was approved by the Institute of Health Studies steering committee. Follow up until 1997 for Cohort 1 and until 1999 for Cohort 2 was achieved through follow up questionnaires and record linkage with MONICA registers, national mortality index register and hospital discharge registers.  <http://www.thl.fi/publications/morgam/cohorts/full/spain/spa-cata.htm> |
| **FINRISK:** The FINRISK study is a series of population-based cardiovascular risk factor surveys carried out every five years in five (or six in 2002) districts of Finland, including North Karelia, Northern Savo (former Kuopio), Southwestern Finland, Oulu Province, Lapland province (in 2002 only) and the region of Helsinki and Vantaa. A stratified random sample was drawn for each survey from the national population register, the age-range was 25-74 years. All individuals enrolled in the study received a physical examination, a self-administered questionnaire, and a blood sample was drawn. In 1997, altogether 11,500 individuals were invited and 8,444 (73%) participated in the clinical examination. During follow-up the National Hospital Discharge Register, the National Causes of Death Register and the National Drug Reimbursement Register were used to identify endpoints. At the moment, the follow-up extends until Dec. 31^st^, 2010, i.e., 14 years for the FINRISK 1997 cohort. The Coordinating Ethics Committee of the Helsinki and Uusimaa Hospital District approved the study, which followed the declaration of Helsinki. All subjects gave written informed consent. <http://www.thl.fi/publications/morgam/cohorts/full/finland/fin-fina.htm> |
| **DAN-MONICA (Glostrup) Study:** The DAN-MONICA cohorts are three prospective population based cohorts from 11 municipalities in the western parts of Greater Copenhagen Area, Denmark. Random sampling based on the national population register, stratified by sex and year of birth was used. Cohort 1 consists of men and women aged 30-70 years. They were examined in 1982-1984 (N=3,785). Cohort 2 was examined in 1987-1988 and cohort 3 of age group 30-70 (N=1,624) was examined in 1991-1992. Follow up is achieved through linkage to the National Cause of Death Register and National Hospital Discharge Register, with endpoint diagnosis based on MORGAM criteria. Follow up for the cohorts 1, 2, and 3 is completed to December 31st 2010. <http://www.thl.fi/publications/morgam/cohorts/full/denmark/den-gloa.htm> |
| **Moli-Sani Project:** The cohort of the Moli-Sani Project was recruited in the Molise region from city hall registries by a multistage sampling. First, townships were sampled in major areas by cluster sampling; then, within each township, participants aged 35 years or over were selected by simple random sampling. Exclusion criteria were pregnancy at the time of recruitment, lack of in understanding, current multiple trauma or coma, or refusal to sign the informed consent. A total of 24,325 men (47%) and women (53%) over the age of 35 were examined at baseline from 2005 to 2010. Participation was 70%. The cohort was followed-up for a median of 4.2 years (maximum 6.5 years) at December 2011.^17^ Follow up is achieved through record linkage to national mortality registries and hospital discharge registers, validation of events was achieved through hospital record linkage and doctors medical records using updated MORGAM criteria. <http://www.moli-sani.org/>  <http://www.thl.fi/publications/morgam/cohorts/full/italy/ita-mola.htm> |
| **The Northern Sweden MONICA Study:** The Northern Sweden MONICA study covered the two northernmost counties of Sweden, i.e. Norrbotten and Västerbotten with altogether 510,000 inhabitants. Population surveys were performed in 1986, 1990, 1994, 1999, 2004 and 2009, with altogether 10,517 unique participants.^18^ On the first two occasions, 2,000 persons aged 25 to 64 years were randomly selected, and in the last three surveys, the upper age limit was extended to 74 years and 2,500 individuals were invited. A stratified randomized selection procedure by age and sex (250 persons in each sex/10-year age stratum) has been used. The participation rate was 69-81%. Detailed analyses of non-participants have been performed. Incident cardiovascular events (myocardial infarction and stroke) occurring in the region between 1985 and 2010 and below the age of 75 were collected and validated according to MONICA criteria by two event registers whose accuracy and validity have been tested against national registers.^19^ Follow-up is available for all cohorts until December 2011 for mortality and non- fatal coronary, stroke, chronic heart failure, atrial fibrillation, cancer and diabetes events. Coronary and stroke events below the age of 75 validated applying the MONICA diagnostic criteria, and diabetes according to careful case review.^20^ , <http://www.thl.fi/publications/morgam/cohorts/full/sweden/swe-nswa.htm> |
| **Prospective Epidemiological Study of Myocardial Infarction (PRIME Belfast) Study:** The PRIME study examined the classic and putative cardiovascular risk factors to explain the large difference in heart disease incidence between Ireland and France.^21^ The study includes four cohorts of men aged 50-59; from Belfast, Northern Ireland (N=2,745) and Lille (N=2,633), Toulouse (N=2,610) and Strasbourg (N=2,612) in France. Baseline examinations took place in 1990-1993 and targeted cohorts which had broadly similar social class structures to the background population, initially sampling from industries and various employment groups, employment groups with more than 10% of their workforce of foreign origin were excluded. Follow up until 2004 (Toulouse, Strasbourg and Lille) and until 2012 (Belfast) was achieved through annual follow up questionnaires with verification against national death registers, medical records, hospital discharge diagnoses. Endpoints were validated by expert medical committee. <http://www.thl.fi/publications/morgam/cohorts/full/uk/unk-bela.htm> |
| **Scottish Heart Health Extended Cohort (SHHEC):** This consists of two overlapping studies which share a common protocol and methods: the Scottish Heart Health Study randomly recruited men and women aged 40-59 across 22 Scottish districts in 1984-1987; Scottish MONICA similarly recruited men and women aged 25-64 in Edinburgh and North Glasgow in 1986, and in North Glasgow again in 1989, 1992 (to 74), and in 1995 as part of the WHO MONICA Project. They are now combined as one cohort, although length of follow-up, currently to the end of 2009, inevitably varies in the different components. Follow up is achieved through flagging for death certificates at the National Health Service Death Register and through the Scottish Record Linkage scheme for deaths and hospital discharge records run by Information Services Scotland, which works on probability matching.^22^ These diagnoses are no longer validated from case notes now that endpoint numbers run into thousands as the cohort ages, but they are allocated to MORGAM categories. Risk factor and endpoint data were used to produce the ASSIGN cardiovascular risk score.^23^ See. <http://www.thl.fi/publications/morgam/cohorts/full/uk/unk-sco.htm> |
| **Disease Cohorts** |
| **Athero*Gene*:** The Athero*Gene* study was conducted at the Department of Medicine II of the University Medical Center Mainz and the Federal Armed Forces Central Hospital Koblenz in Germany from 1996 to 2004.^24^ The major goal of the Athero*Gene* study was to assess risk prediction across a wide range of coronary artery disease phenotypes with particular emphasis on the role of genetics. The major endpoint in this study was cardiovascular death and non-fatal myocardial infarction among other cardiovascular outcomes. Inclusion criteria for this study were prevalent coronary artery disease with angiographical diagnosis of a 30% or higher stenosis in a major coronary artery. Exclusion criteria were evidence of hemodynamically significant valvular heart disease, surgery or trauma within the previous month, known cardiomyopathy, malignancies, febrile conditions, chronic inflammatory diseases, renal failure (increased creatinine >2.1 mg/dL) or use of oral anticoagulant therapy within the previous four weeks. Patients presenting with solely elevated C-reactive protein concentrations were not excluded from the study cohort. Fasting blood was drawn prior to catheterization in the cath lab and processed immediately. |
| **Langzeiterfolge der Kardiologischen Anschlussheilbehandlung (KAROLA):** KAROLA is an observational, prospective cohort study in which 1,206 patients with coronary heart disease (CHD) aged 30-70 years participating in an in-hospital rehabilitation program between January 1999 and May 2000 in two co-operating clinics (Schwabenland-Klinik, Isny and Klinik am Südpark, Bad Nauheim, Germany) were enrolled. Only patients who were admitted within 3 months after their first acute event or coronary artery revascularization were included. Baseline data included detailed information from medical records, medical exams and patient questionnaires. An active follow-up has been conducted 1, 3 and 4.5, 6, 8, 10 and 13 years later.^25, 26^ Beside life style factors, quality of life and other patient reported information obtained by contacting the patient, the occurrence of secondary cardiovascular disease events was evaluated by contacting the primary care physicians. If a subject died during follow-up, the death certificate was obtained from local Public Health departments and the main cause of death was coded according to the International Classification of Diseases. Follow-up is ongoing. |
| **Study for evaluation of newly onset chest pain and rapid diagnosis of myocardial necrosis (steno*Cardia*):** Patients with acute chest pain presenting consecutively at the chest pain unit of the Johannes Gutenberg-University Medical Centre Mainz between January 2007 and December 2008 were enrolled in this all-comers prospective biomarker assessment registry.^27, 28^ Blood samples were obtained on admission and after 3 and 6 hours. Routine laboratory parameters including C-reactive protein were measured immediately after blood withdrawal by standardized methods. Additionally, EDTA plasma and serum samples were collected at each time point, centrifuged, aliquoted and stored at -80°C. The study was approved by the local ethics committees. Participation was voluntary; each patient gave written, informed consent. |
